# Nanopatterned Monolayers of Bioinspired, Sequence-Defined Polypeptoid Brushes for Semiconductor/Bio Interfaces

**DOI:** 10.1021/acsnano.3c10204

**Published:** 2024-02-27

**Authors:** Beihang Yu, Boyce S. Chang, Whitney S. Loo, Scott Dhuey, Padraic O’Reilly, Paul D. Ashby, Michael D. Connolly, Grigory Tikhomirov, Ronald N. Zuckermann, Ricardo Ruiz

**Affiliations:** † The Molecular Foundry, Lawrence Berkeley National Laboratory, Berkeley, California 94720, United States; ‡ Prizker School of Molecular Engineering, University of Chicago, Chicago, Illinois 60637, United States; § Molecular Vista Inc., San Jose, California 95119, United States; ∥ Department of Electrical Engineering and Computer Sciences, University of California, Berkeley, Berkeley, California 94709, United States

**Keywords:** nanopatterned polymer brushes, sequence-defined polymers, semiconductor/bio interfaces, surface modification, selective immobilization of biomolecules

## Abstract

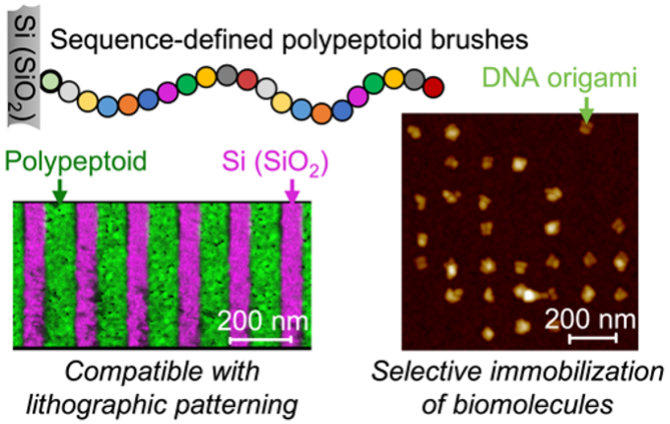

The ability to control and manipulate semiconductor/bio interfaces is essential to enable biological nanofabrication pathways and bioelectronic devices. Traditional surface functionalization methods, such as self-assembled monolayers (SAMs), provide limited customization for these interfaces. Polymer brushes offer a wider range of chemistries, but choices that maintain compatibility with both lithographic patterning and biological systems are scarce. Here, we developed a class of bioinspired, sequence-defined polymers, i.e., polypeptoids, as tailored polymer brushes for surface modification of semiconductor substrates. Polypeptoids featuring a terminal hydroxyl (−OH) group are designed and synthesized for efficient melt grafting onto the native oxide layer of Si substrates, forming ultrathin (∼1 nm) monolayers. By programming monomer chemistry, our polypeptoid brush platform offers versatile surface modification, including adjustments to surface energy, passivation, preferential biomolecule attachment, and specific biomolecule binding. Importantly, the polypeptoid brush monolayers remain compatible with electron-beam lithographic patterning and retain their chemical characteristics even under harsh lithographic conditions. Electron-beam lithography is used over polypeptoid brushes to generate highly precise, binary nanoscale patterns with localized functionality for the selective immobilization (or passivation) of biomacromolecules, such as DNA origami or streptavidin, onto addressable arrays. This surface modification strategy with bioinspired, sequence-defined polypeptoid brushes enables monomer-level control over surface properties with a large parameter space of monomer chemistry and sequence and therefore is a highly versatile platform to precisely engineer semiconductor/bio interfaces for bioelectronics applications.

Harnessing the spatial resolution and positioning accuracy of nanopatterning technology with the programmability and precision of biomacromolecules has the potential to enable technologies for fundamental biophysical research and clinical bioelectronic devices. These devices such as biosensors and disease diagnostic and treatment implants,^1−5^ benefit from well-defined surface nanopatterns, offering superior single-molecule resolution and sensitivity compared to homogeneous or micropatterned surfaces. For example, optical nanostructures such as zero-mode waveguides with selective immobilization of enzyme molecules in the confined subdiffraction observation volume enable single-molecule investigation of enzymatic activities at biologically relevant concentrations.^6,7^ Nanoscale features on material surfaces (topographical and chemical cues) can program cell adhesion and fate, offering fundamental biological insights into the cell behavior in response to their microenvironment. These insights are crucial for advancing cell culture materials and regenerative medicines including implants and stem cell therapeutics.^8,9^ The successful development of bioelectronic or optoelectronic devices interfacing with biological systems with single-molecule resolution will require precise design and customization of surface modification layers with exact nanoscale patterning at the inorganic/bio interface. These surface modification layers, which consist of molecular coatings in direct contact with biological systems, enable the viability of the interface by fulfilling various functions. They can adjust surface hydrophilicity or hydrophobicity, facilitate molecular immobilization or antifouling in specific regions, regulate short-range interactions, and even provide specific molecular recognition.

Fabricating functional and biocompatible nanointerfaces is challenging due to the inherent complexity of biological systems and the need for versatile, customizable surface modification materials that can effectively bridge inorganic and biological surfaces at the nanoscale. These materials must meet three main criteria: (1) versatile interfacial interactions: the surface modification material should enable a broad spectrum of customizable interactions with biomolecules, ranging from repulsion or antifouling properties to various levels of preferential attachment and specific molecular recognition; (2) substrate compatibility: the surface modification should be uniformly thin and applicable to a variety of substrates, including dielectric, semiconductor, and metallic surfaces; (3) compatibility with lithographic processes: the surface modification material should integrate into lithographic flows, and it must withstand common lithographic processes including exposure to organic solvents, resist materials, UV or electron radiation, and high-temperature baking, without compromising its chemical properties and biocompatibility.

Self-assembled monolayers (SAMs)^10−16^ and polymer brushes^10,17−29^ are perhaps the most commonly used surface modification materials. They have driven many of the recent advances in biosensing and (opto)bioelectronic devices. The molecular structure of SAMs includes a headgroup to attach the molecule to the surface, a backbone that provides structure or self-organization, and a tail group that defines surface energy or provides a site for biomolecular binding.^16^ For example, silane-based and thiol-based monolayers are the most common SAM motifs utilized on oxide and noble metal substrates, and a variety of functionalized SAMs are routinely patterned for applications in biological assays and for cell attachment surfaces.^11,14^ However, in some cases, SAMs suffer from instabilities or excess reactivities in the attachment chemistry which lead to nonuniform modification layers.^13^ Polymer brushes provide a higher level customization with a wider range of chemistry, more controllable surface coverage, and more uniform layer thickness. Yet, as the spatial resolution requirements and complexity of biointerfaces progress, strategies using traditional polymer brushes face challenges. Traditional polymer brushes lack the versatility to incorporate various chemical functionalities required by different biointerfacing needs. Random and block copolymer brushes have been reported, yet in most cases, only two or three monomers are utilized,^24,27^ which may not fulfill the requirements of biointerfaces to conveniently tune coupled surface properties such as wettability and surface presence of chemical groups. Polymer brush layers prepared via the “grafting from” method typically produce relatively thick films, often reaching tens of nanometers or more.^27^ This thickness limitation is problematic when close proximity of the biomaterial to the inorganic surface is important, as in gated thin-film transistor or thin topographic structures like zero-mode waveguides.^4,7^ In summary, there is still a need for versatile, customizable surface modification materials to expand our ability to tune and control a wide variety of interactions at inorganic/bio interfaces that are compatible with high-resolution, high-precision lithographic patterning while maintaining their compatibility with biological systems.

Here, we introduce a family of surface modification materials based on bioinspired, sequence-defined polypeptoid brushes, which offer an ideal means to bridge the gap between synthetic and biomaterials. There is also a need to bridge the gap between small molecule SAMs that have precise molecular structures and higher molecular weight yet disperse polymers. Polypeptoids, or poly(*N*-substituted glycine)s,^30,31^ share similarities with polypeptides but offer better solubility and processability in a range of organic solvents and increased resistance to thermal and protease degradation.^32−35^ They can be precisely synthesized with defined sequences for tailored molecular structures and functionalities. Furthermore, polypeptoids feature highly versatile and diverse side chains,^31,36^ which can range from enabling compatibility with inorganic materials and processes to ensuring compatibility and stability with biomaterials. We designed and synthesized polypeptoids with a terminal hydroxyl (−OH) group that reacts with the activated surface with silanol groups^37,38^ to graft the polymer onto Si substrates and form ∼1 nm thick tethered brush monolayers, which enable efficient modification of the surface properties. This work is organized as follows: In the first section, we detail the design and structure of five polypeptoids of different compositions and sequences, along with the process for creating uniform brush monolayers grafted on Si substrates. The second section focuses on the versatility of the polypeptoid brush platform, highlighting its capability to customize and fine-tune a wide range of interfacial interactions. This includes (1) modulating surface energy, demonstrated by achieving a range of water contact angles; (2) achieving passivation and selective immobilization of biomolecules through various short-range interactions, particularly in relation to DNA origami nanostructures; (3) enabling specific recognition and binding of biomolecules at surfaces via biotin–streptavidin interactions. In the final section of the paper, we demonstrate compatibility of the polypeptoid brushes with lithographic processes to generate nanoscale patterns offering high-resolution chemical contrast and diverse chemical functionalities. We utilized these nanopatterned surfaces to achieve highly selective and localized immobilization of DNA origami nanostructures or streptavidin proteins.

## Results and Discussion

### Grafting Polypeptoid–OH onto Si Substrates as Surface Modification Layers

**Figure 1:**
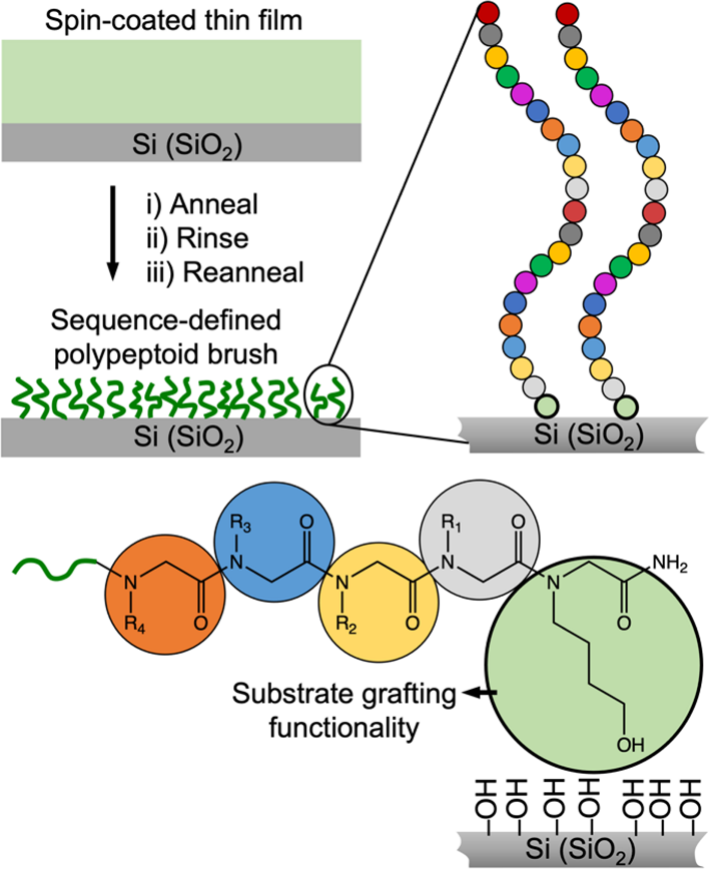
Preparation of grafted polypeptoid brush monolayers on Si substrates via the “grafting to” approach with a hydroxyl (−OH) group as the substrate grafting functionality incorporated on the first monomer at the C-terminus.

Surface-tethered polymer brushes created through the “grafting from” method are known for forming denser monolayers compared to the “grafting to” method.^24,29,39,40^ However, the latter eliminates the need for *in situ* polymerization and offers broader substrate compatibility while still delivering effective surface modification as long as coverage is uniform at relevant molecular scales. Here, we choose the convenience of the “grafting to” method. The polypeptoids feature a hydroxyl (−OH) group on the side chain of the first monomer at the C-terminus (Figure 1), providing strong binding to oxide surfaces like activated SiO_2_ with silanol groups.^37,38^ Five hydroxyl-terminated polypeptoid 21-mers (PP**1** through PP**5**, Table 1) were designed and synthesized with a combination of polar and nonpolar monomers to adjust hydrophilicity and other properties impacting processability, such as solubility in organic solvents and crystallization inhibition for spin coating, as well as compatibility with liquid chromatography characterization.

**Table 1:**
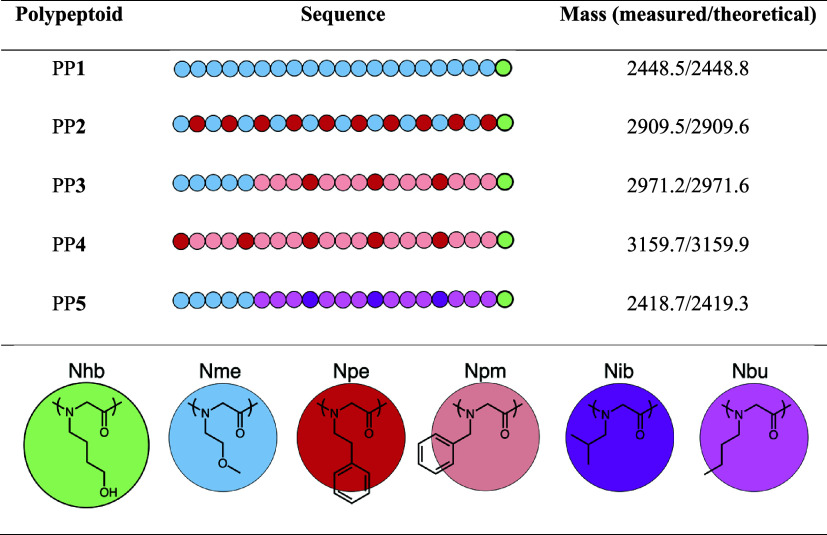
Polypeptoids Synthesized for This Study

**Figure 2:**
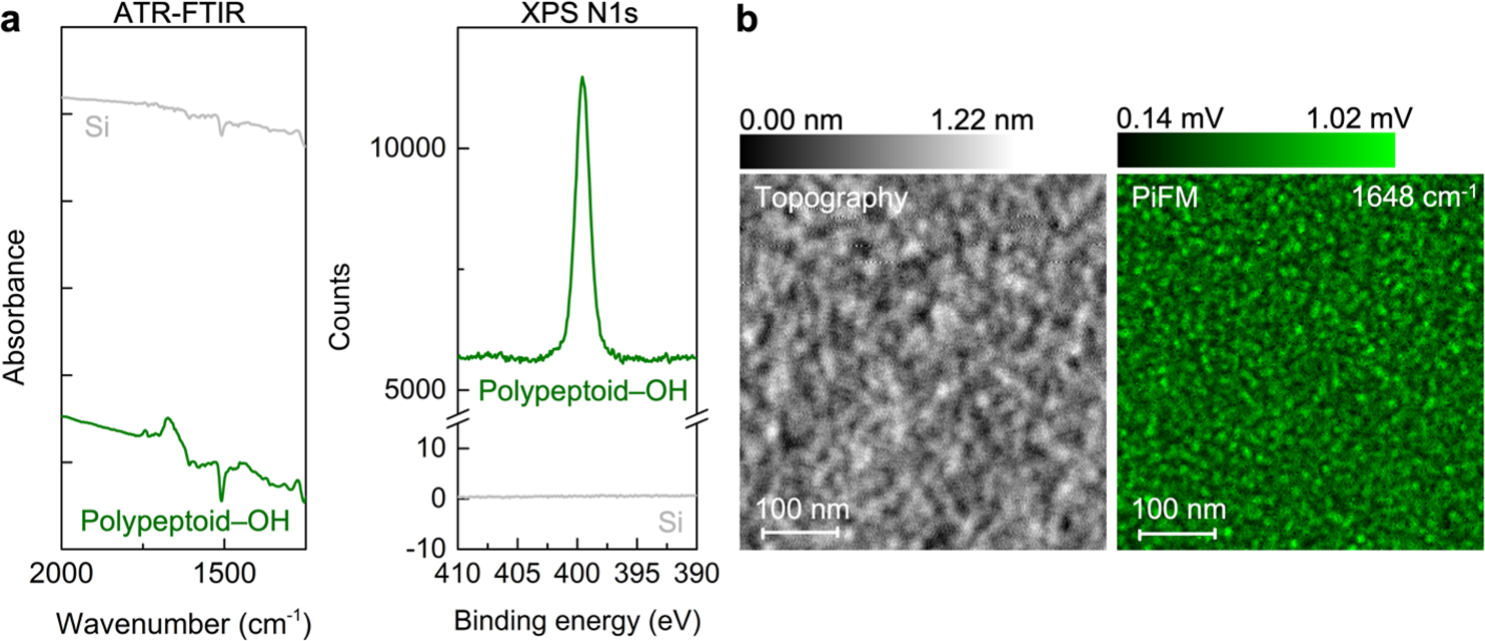
(a) ATR-FTIR and XPS N 1s spectra of prepared polypeptoid brush monolayers on Si substrates. The presence of an IR band of ∼1670 cm^–1^ for polypeptoid amide C=O stretching and a N 1s peak between 390 and 400 eV confirms successful grafting of −OH functionalized polypeptoids. (b) Topography and PiFM (mapped at 1648 cm^–1^, corresponding to the amide C=O stretching of polypeptoids) characterization indicates good surface coverage of the substrate by the grafted polypeptoid brush monolayer with a smooth top surface.

The polypeptoid 21-mers used in this study have molecular weights between 2400 and 3200 g mol^–1^ (Table 1), which would have a radius of gyration (*R*_g_) of ∼1 nm based on previous small-angle neutron scattering (SANS) measurements of similar polypeptoids in near-θ conditions.^41,42^ With the potentially very thin brush monolayers, multiple surface characterization techniques are employed to confirm the surface coverage of Si substrates with grafted polypeptoids. With attenuated total reflectance-Fourier transform infrared (ATR-FTIR) spectroscopy and X-ray photoelectron spectroscopy (XPS), the presence of grafted polypeptoids on substrates is confirmed by the amide C=O stretching peak at ∼1670 cm^–1^ in ATR-FTIR, and the N 1s peak between 390 and 400 eV in XPS (Figure 2a). Yet, these two macroscopic characterization techniques do not provide direct evidence on whether homogeneous modification of the substrate surface on nanoscopic length scales is achieved.

In order to examine the surface coverage at nanoscale resolution, we further use infrared photoinduced force microscopy (IR PiFM) to directly visualize the surface coverage of Si substrates by grafted polypeptoids. IR PiFM is a nanoscale microscopy and spectroscopy technique that measures photoinduced thermal response and polarizability of samples in the near field by detecting the force between the tip and the sample. This technique enables simultaneous spatial mapping of topographical and chemical information from two mechanical eigenmode resonances of the cantilever at the resolution of an atomic force microscope (AFM).^43−45^ The prepared polypeptoid brush monolayers are topographically smooth, with a root-mean-square roughness of *R*_q_ ≈ 0.2 nm, comparable to bare Si substrates. Importantly, the chemical map collected simultaneously at 1648 cm^–1^ (characteristic band of polypeptoid backbone amide C=O stretching) indicates that the substrate is well covered with a grafted polypeptoid brush monolayer (Figure 2b). These results indicate the feasibility of achieving uniform coverage and, notably, uniform chemical modification using the “grafting to” method, at least down to the resolution of an AFM (a few nanometers in lateral resolution), despite the lower grafting density (σ) characteristic of the “grafting to” approach. Further estimation of the value of σ will be discussed later in the manuscript. While both carboxylic acid (−COOH) and hydroxyl (−OH) functionalities have been reported in literature as functional groups for grafting polymers to the native oxide layer of Si substrates under melt grafting conditions,^19,46−52^ here, we found that, under the same melt grafting conditions, i.e., annealing polypeptoid thin films spin coated on Si substrates at 180 °C for 30 min, only the −OH functionalized polypeptoids efficiently graft onto Si substrates, while the −COOH functionalized polypeptoids only form discrete aggregates on the equivalently treated substrates (Figure S8). Similar trends in grafting efficiency between PS–OH and PS–COOH and PMMA–OH and PMMA–COOH are observed under the same melt grafting conditions (Table S1). Condensation reactions between carboxylic acids and free silanol groups have been reported under acetic conditions (pH = 2) with a 0–15% coupling efficiency.^53^ Without the presence of an acid in solution, molecules with carboxylic acid groups may only physisorb on silicon oxide surfaces via hydrogen bonds.^54,55^ We suspect that our conditions with thermal annealing are insufficient for the −COOH functionalized polymers to form stable bonds with surface silanol groups as compared to −OH functionalized polymers.

### Customization in Surface Modification Enabled by Polypeptoid Brushes

The ultrathin polypeptoid brush monolayers are excellent surface modification materials because they can be used to controllably tune the surface properties of Si substrates. Using polypeptoids with a terminal hydroxyl group, we demonstrate that, through designing monomer chemistry and composition, different levels of complexity in modifying surface properties can be achieved: from the simplest surface energy, to affinity toward biomolecules from passivation to preferential attachment, and further to specific binding of biomolecules.

#### Surface Hydrophilicity and Surface Free Energy

**Figure 3:**
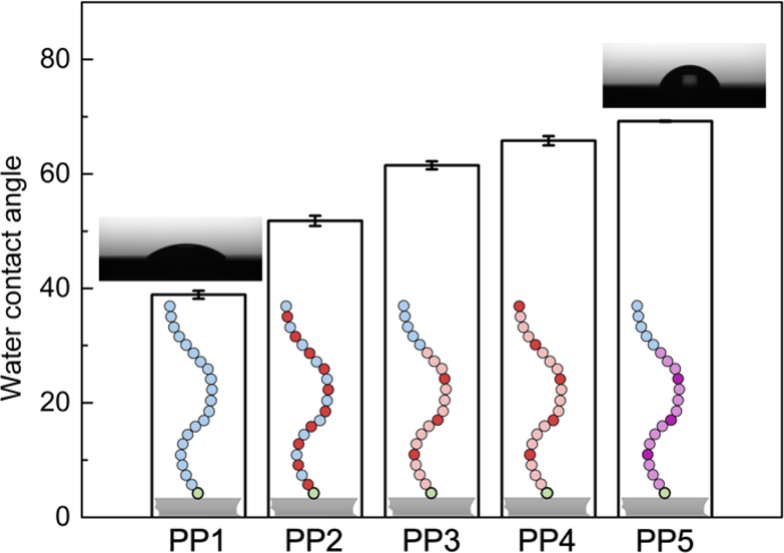
Surface hydrophilicity of Si substrates, as evidenced by different static water contact angles, is modified accordingly by the grafted polypeptoid brush monolayers, depending on the monomer chemistry and monomer composition. See Table 1 for chemical structures of the polypeptoids.

With the grafted brush monolayers of PP**1**–**5**, the surface hydrophilicity of Si substrates can be modified accordingly as evidenced by the static water contact angle ranging from 38.9 ± 0.7° to 69.2 ± 0.1° (Figure 3). With the amide backbone, polypeptoids are polar in nature, yet by introducing side chains of different polarities, the overall polarity of the polymer can be tuned. It is expected that polar side chains (e.g., the methoxyethyl group) would lead to more hydrophilic surfaces and surfaces with a higher polar contribution in the surface free energy. It is also expected that nonpolar aromatic and alkyl groups would lower the overall polarity of the polypeptoid. Indeed, polypeptoid brushes with ≥50% of nonpolar side chains lead to much higher water contact angles and a very small polar contribution in the surface free energy, as compared to PP**1** modified surfaces (Table S2). Here, we note the polypeptoid brush modified surfaces are not hydrophobic; however, it is possible to further expand the tunable range of surface hydrophilicity and surface free energy with grafted polypeptoids by introducing more nonpolar side chains such as longer alkyl or fluorinated side chains.^56−59^

#### Passivation and Preferential Attachment of Biomolecules

**Figure 4:**
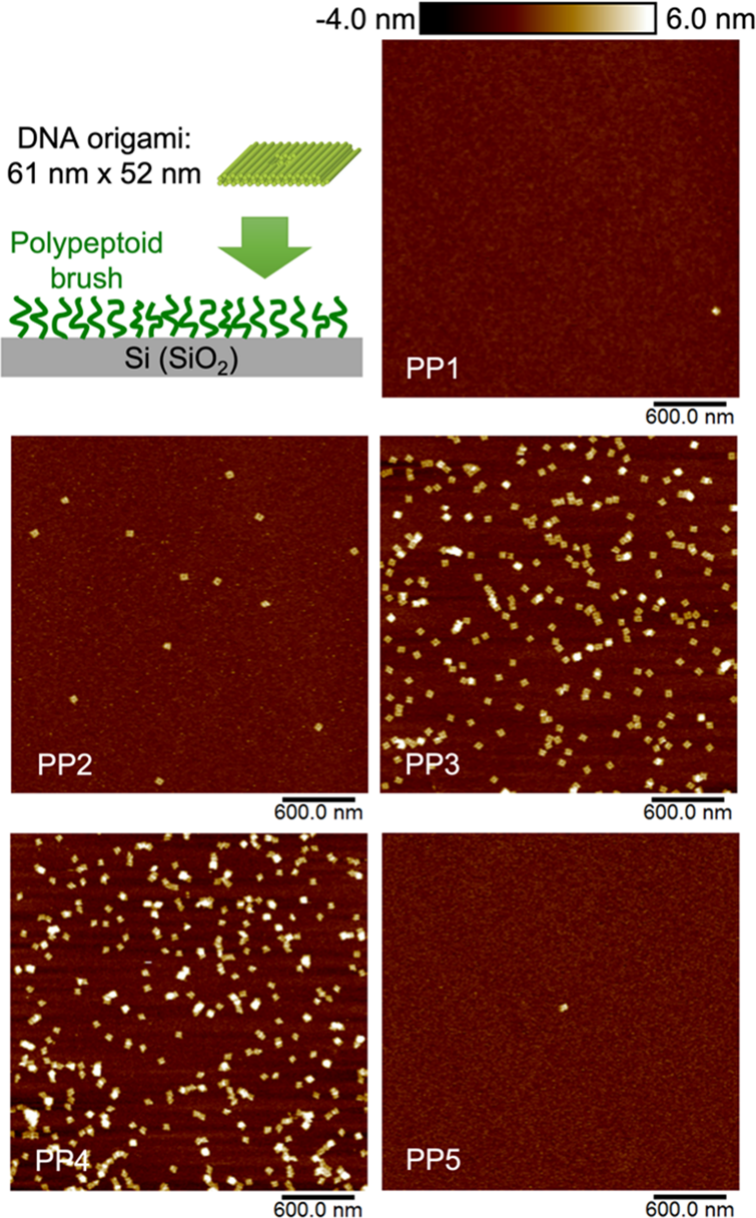
Si substrates modified with different polypeptoid brush monolayers exhibit tunable affinity from passivation to preferential attachment of DNA origami nanostructures.

Tunable affinity toward biomolecules is a highly desired property of surfaces. Here, we demonstrate that polypeptoid brush monolayers are capable of tuning the affinity of surfaces toward DNA origami nanostructures (61 × 52 × 8 nm cuboid nanostructure with an aperture). Among the five polypeptoids, two polypeptoids are identified as good surface passivation molecules with either methoxyethyl or butyl side chains, while polypeptoids containing aromatic groups induce preferential attachment of DNA origami nanostructures on corresponding surfaces (Figure 4). Previous strategies for surface passivation against DNA origami are commonly achieved with a hydrophobic trimethylsilyl layer produced by hexamethyldisilane (HMDS).^60−63^ The binding of DNA origami on surfaces is most commonly mediated through electrostatic interactions, using Mg^2+^ as the electrostatic bridge between negatively charged DNA origami and negatively charged surfaces (e.g., Si, mica).^60,64^ Here, we discovered that the binding affinity of DNA origami is decoupled from the surface hydrophilicity. Both PP**1** and PP**5** modified surfaces exhibit minimal affinity toward DNA origami, while these two surfaces are the most and the least hydrophilic (water contact angle: 38.9 ± 0.7° vs 69.2 ± 0.1°) among the five polypeptoid brush monolayer modified surfaces. PP**3** and PP**4** with a high aromatic monomer composition (75% and 100%, respectively) enable preferential attachment of DNA origami on surfaces, with a much higher density compared to the commonly used activated Si substrates under the same deposition conditions (Figure S11). The results indicate the possibility of aromatic groups driving DNA origami attachment on surfaces, yet in this study, we do not attempt to further elucidate the underlying molecular mechanism.

#### Specific Binding of Biomolecules

**Figure 5:**
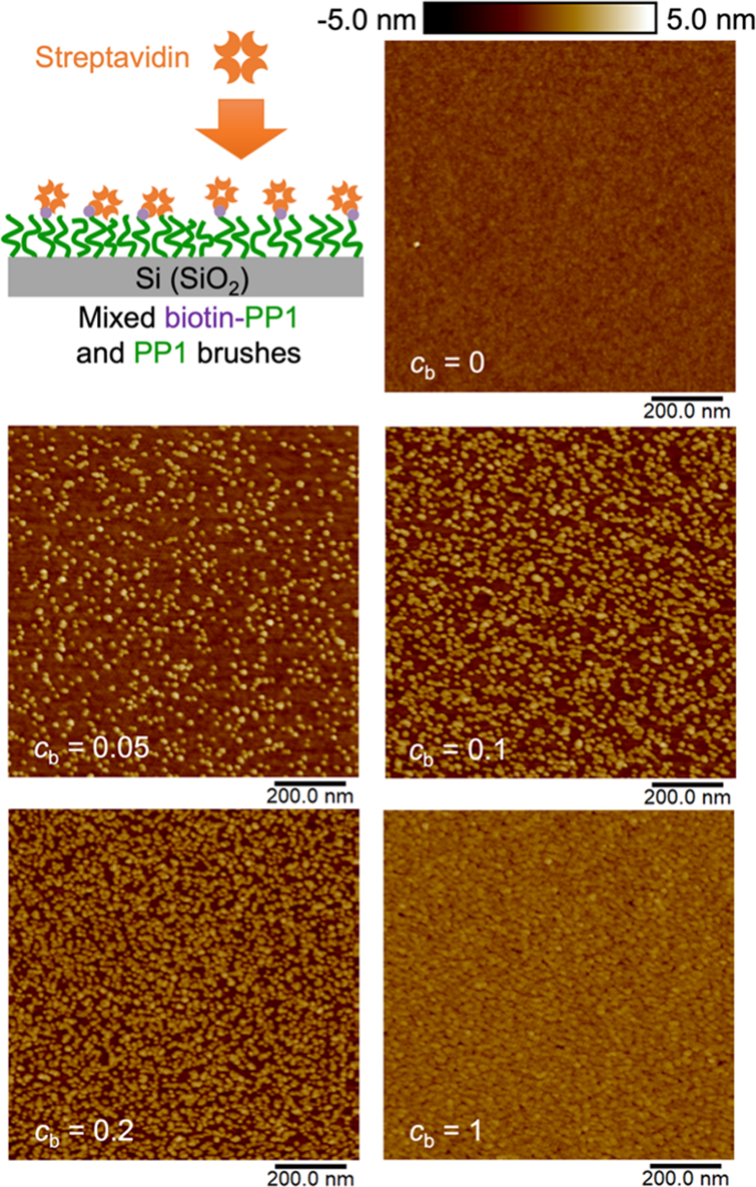
Immobilization of streptavidin via specific biotin–streptavidin interactions at different binding densities on surfaces, achieved by controlling surface biotin group density through grafting biotin-PP**1** and PP**1** mixtures (relative concentration of biotin-PP**1** in PP**1**, *c*_b_) onto Si substrates.

Specific protein binding events typically involve biomolecular recognition between specific chemical groups and the protein. Here, we show that as a surface modification material, functionalized polypeptoid brushes enable specific binding of proteins with a demonstration based on biotin–streptavidin interactions. PP**1** is first identified to generate modified surfaces with minimal nonspecific binding of streptavidin (Figure S12). This is within expectation as polypeptoid brushes with methoxyethyl side chains have been reported in multiple studies for creating antifouling surfaces against lysozyme, fibrinogen and serum proteins, fibroblast cells, and bacteria.^65−67^ Next, biotinylated PP**1** is synthesized by biotinylation of the N-terminus as the last step during solid-phase synthesis (Scheme S2). Therefore, the immobilization of streptavidin on the biotin-PP**1** modified surface is enabled by the specific biotin–streptavidin interactions, where nonspecific adsorption of streptavidin is minimized. As shown in Figure 5, different streptavidin binding density on surfaces is achieved through tuning the relative concentration of biotin-PP**1** in PP**1**, *c*_b_, and then grafting the polypeptoid mixtures onto Si substrates (streptavidin fractional surface coverage, φ, is quantified and plotted as a function of *c*_b_ in Figure S13).

### Nanoscale Surface Patterns of Polypeptoid Brushes

#### Compatibility of Polypeptoid Brushes with Lithographic Nanopatterning

**Figure 6:**
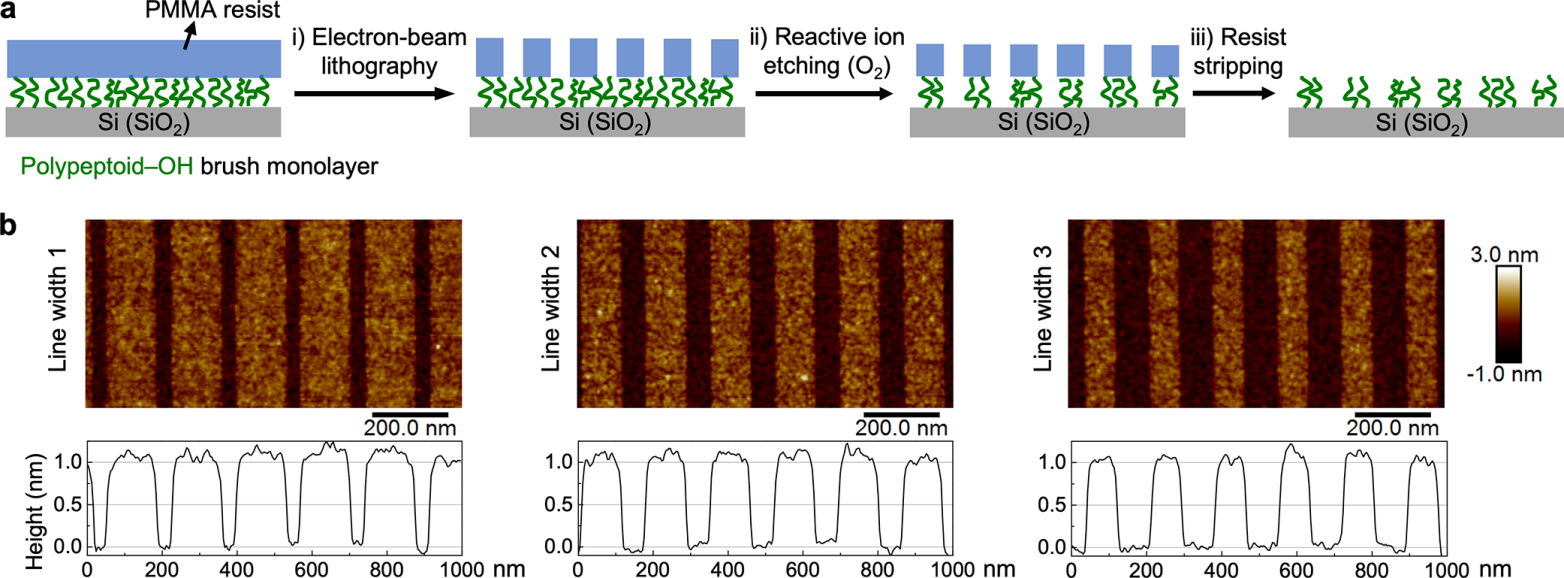
(a) Schematic of the lithographic patterning workflow to pattern a polypeptoid brush monolayer grafted on Si substrate: (i) electron-beam lithography to pattern the spin-coated PMMA resist layer on top of the brush monolayer, (ii) reactive ion etching with oxygen plasma to transfer the pattern into the underlying polypeptoid brush monolayer, and (iii) stripping the PMMA resist and reannealing the patterned polypeptoid brush monolayer. (b) AFM topography images of generated line-space patterns (pitch = 170 nm) with different line widths, from which the height profiles show the polypeptoid brush monolayer is ∼1 nm in thickness.

The compatibility with lithographic patterning of the polypeptoid brush monolayers is an important attribute of this class of bioinspired, sequence-defined polymers as surface modification materials as it will enable the generation of chemical contrast nanopatterns by design with monomer-level control. The lithographic patterning workflow adopted in this study follows a typical strategy of patterning surface modification layers on Si substrates.^52,61,68,69^ As shown in Figure 6a, it involves the following steps: (i) spin coating an ∼40 nm PMMA resist on top of the polypeptoid brush monolayer for electron-beam lithography, with a subsequent development step; (ii) reactive ion etching with oxygen plasma to transfer the pattern of the PMMA resist layer into the underlying polypeptoid brush monolayer; (iii) stripping the PMMA resist and reannealing the polypeptoid brush to obtain a nanopatterned polypeptoid brush monolayer on Si substrates. AFM height profiles show that the generated line-space patterns are well-defined with sharp edges and reveal that the polypeptoid brush monolayers are ∼1 nm in thickness (Figure 6b). With the monolayer thickness obtained from nanopatterned polypeptoid brushes, the estimated grafting density (σ) based on the formula  is ∼0.24 chain nm^–2^, where ρ is the polypeptoid density (taken as 1.2 g cm^–3^ based on reported values in the literature),^70−72^*N*_A_ is Avogadro’s number, *d* is the brush thickness, and *M*_w_ is the molecular weight of polypeptoids (here, the nanopatterned polypeptoid brush is PP**3** with a *M*_w_ of 2971 g mol^–1^). As aforementioned, these polypeptoid 21-mers should have a radius of gyration (*R*_g_) of ∼1 nm and a contour length of ∼7 nm based on previous SANS measurements of similar polypeptoids in close to θ-conditions.^41,42^ With the brush thickness comparable to *R*_g_, it suggests that the polypeptoid brush grafting density is relatively low, possibly in a “mushroom” regime close to an overlap density, as the brush top surface still appears smooth. Using the “mushroom” to “brush” transition (Σ = σπ*R*_g_^2^ ∼ 1),^39,73,74^ it suggests the polypeptoid grafting density is near or below this transition σ ∼ 0.26 chain nm^–2^, which is consistent with the earlier estimation. A relatively low grafting density is common in brushes prepared via the “grafting to” method.^24,40^

**Figure 7:**
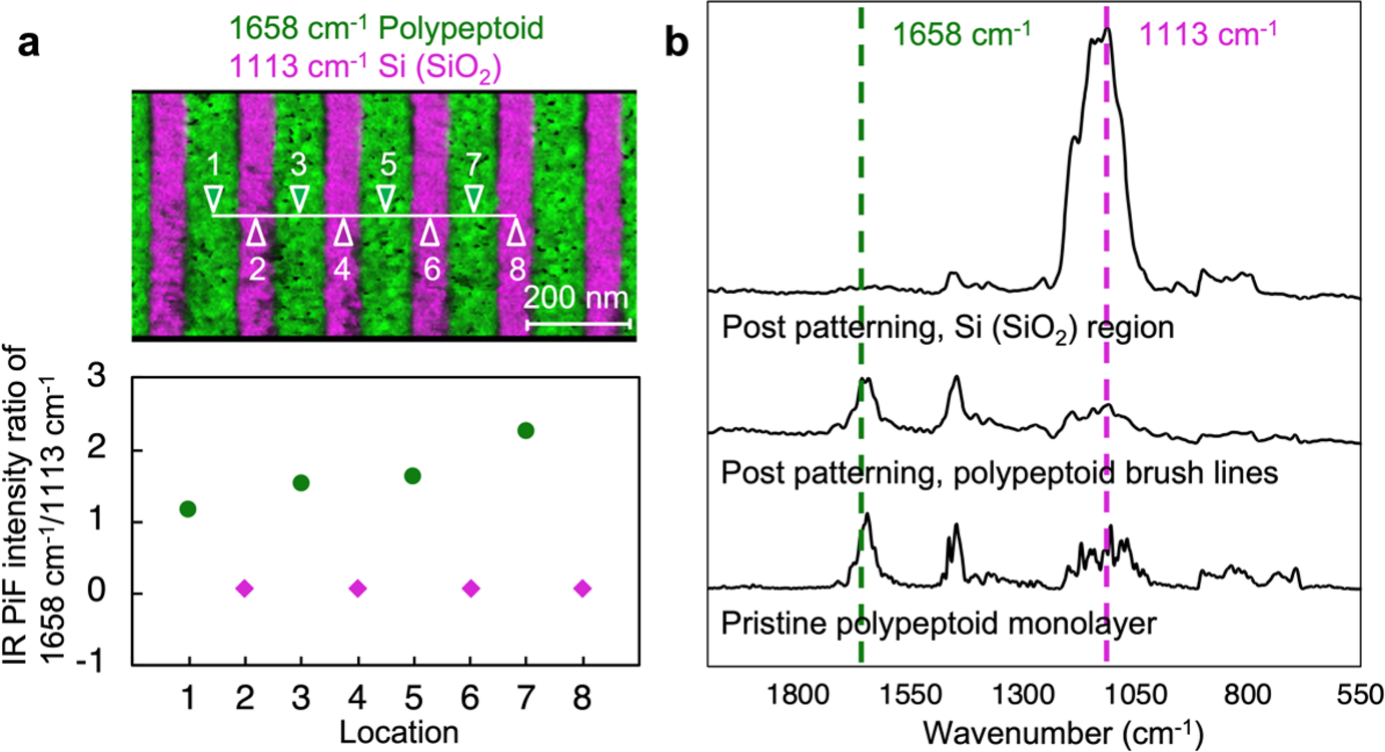
(a) Combined PiFM image mapped at 1658 and 1113 cm^–1^, corresponding to amide C=O stretching of polypeptoids and Si–O–Si stretching of SiO_2_, respectively, demonstrates the successful electron-beam lithographic patterning of polypeptoid brush monolayers grafted on Si substrates. The PiF-IR signal intensity ratios of 1658 cm^–1^/1113 cm^–1^ at locations on Si (SiO_2_) trenches (locations 2, 4, 6, and 8) are near zero; i.e., trenches are clean with unobservable polypeptoid residues. (b) From bottom to top: averaged PiF-IR spectrum of 6 locations on a pristine polypeptoid brush monolayer before electron-beam lithography, averaged PiF-IR spectrum of locations 1, 3, 5, and 7 on the polypeptoid brush lines generated by electron-beam lithography, averaged PiF-IR spectrum of locations 2, 4, 6, and 8 on Si (SiO_2_) trenches post-electron-beam lithographic patterning. The similar PiF-IR spectra of the pristine polypeptoid brush monolayer and nanopatterned polypeptoid brush lines indicate that the chemical characteristics of this polypeptoid brush-based system are preserved through the lithographic workflow.

To further probe and confirm the chemical characteristics of polypeptoid brush monolayers post-lithographic patterning, the nanopatterned brush monolayers are mapped with IR PiFM at two wavenumbers, 1658 and 1113 cm^–1^, with the former corresponding to the amide C=O stretching of polypeptoids and the latter corresponding to the Si–O–Si stretching of the native oxide layer at the Si substrate surface. The combined PiFM image shows the line-space patterns are well-defined chemically, with the Si (SiO_2_) trenches with unobservable polypeptoid residues, as indicated by the near-zero PiF-IR signal intensity ratio of 1658 cm^–1^/1113 cm^–1^ at locations 2, 4, 6, and 8 (Figure 7a). More importantly, comparing the averaged full PiF-IR spectrum of sampled locations on the nanopatterned polypeptoid brush monolayer (locations 1, 3, 5, and 7) and the spectrum of a pristine polypeptoid monolayer before lithographic patterning, no distinct difference is observed between the two PiF-IR spectra, with the characteristic peak of polypeptoid amide C=O stretching at ∼1660 cm^–1^ clearly observed post-electron-beam lithographic patterning (Figure 7b). This evidence indicates that the chemical characteristics of this polypeptoid brush-based system are preserved through the lithographic workflow which involves harsh conditions including background electron-beam radiation, oxygen plasma etching, and various organic solvents used in the process.

#### Generation of Chemical Contrast Nanopatterns Consisting of Two Alternating Polymer Brushes

Generation of robust, well-defined chemical contrast patterns at desired resolution is of critical importance for applications such as biological assays for sensing and diagnostics,^11,75^ surfaces for cell adhesion and growth,^76,77^ directed self-assembly of block copolymers,^52,68,69,78^ and site-specific immobilization of nanoscale objects such as DNA origami nanostructures and gold nanoparticles.^19,60−62^ In particular, leveraging different polymer brushes to modify the corresponding lithographically defined regions is a common strategy to generate chemical contrast nanopatterns, where the effects from potential brush interpenetration could be minimized by molecular weight engineering per specific application requirements.^50−52^ With a sequence-defined polymer brush platform, the parameter space of chemical contrast nanopatterns can be further expanded with precise, monomer-level engineering of the chemical functionalities.

**Figure 8:**
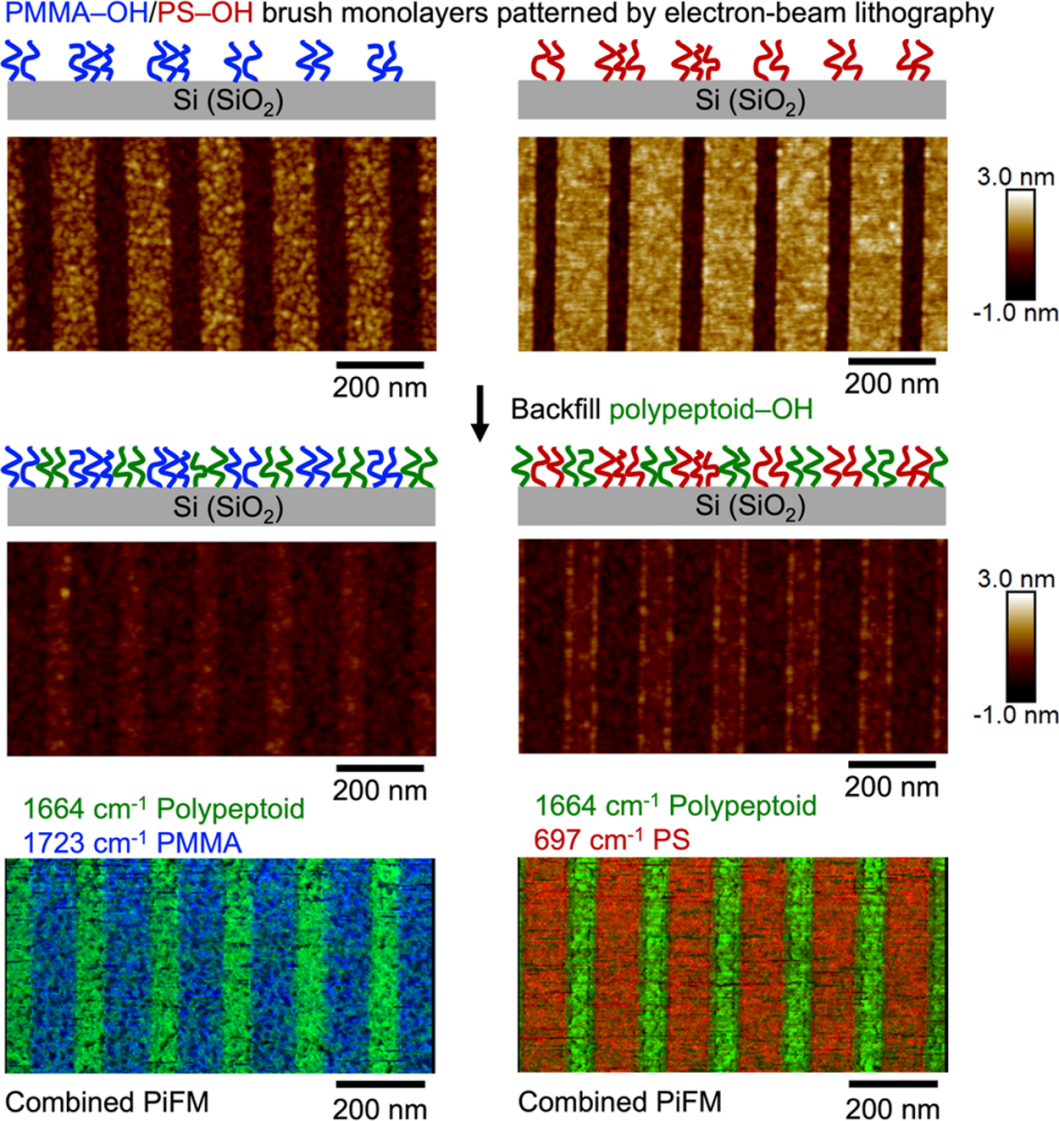
Surface chemical contrast nanopatterns consisting of alternating lines of two polymer brushes are generated by patterning a first polymer brush monolayer (e.g., PMMA–OH or PS–OH) with electron-beam lithography and then backfilling a second polymer (polypeptoid–OH) to graft onto the exposed Si (SiO_2_) surface. The combined PiFM images mapped at wavenumbers corresponding to the characteristic bands of each polymer (polypeptoid, 1664 cm^–1^; PMMA, 1723 cm^–1^; PS, 697 cm^–1^) confirm the chemical characteristics of the generated surface nanopatterns.

To generate chemical contrast nanopatterns with −OH terminal functionalized polymers, we demonstrate a workflow by nanopatterning a first polymer brush monolayer grafted on a Si substrate, followed by backfilling a second −OH functionalized polymer brush to graft onto the exposed Si (SiO_2_) trenches, to form chemical contrast nanopatterns that consist of two polymer brushes. As shown in Figure 8, a PS–OH or PMMA–OH brush monolayer is first nanopatterned by electron-beam lithography to generate PS–Si (SiO_2_) or PMMA–Si (SiO_2_) line-space nanopatterns, followed by backfilling a polypeptoid–OH brush. The successful grafting of −OH functionalized polypeptoids is evidenced by the topographical changes observed with AFM after the polypeptoid–OH backfill step, where the Si (SiO_2_) trenches are grafted with polypeptoid brushes, resulting in almost coplanar PS–polypeptoid or PMMA–polypeptoid nanopatterns. The chemical characteristics of the generated nanopatterns of alternating PS (or PMMA) and polypeptoid brush line patterns are further confirmed by IR PiFM mapping at the characteristic bands for polypeptoid (1664 cm^–1^), PMMA (1723 cm^–1^), and PS (697 cm^–1^), respectively (Figure 8). Notably, while both polypeptoid and PMMA have carbonyl groups, the C=O stretching bands of the two polymers show up ∼60 cm^–1^ apart (peak at 1664 cm^–1^ for amide C=O stretching in polypeptoid, peak at 1723 cm^–1^ for ester C=O stretching in PMMA) in the full PiF-IR spectra collected between 2000 and 541 cm^–1^ (Figure S9), well within the tool resolution limit to clearly distinguish between the grafted PMMA and polypeptoid brushes of ∼1 nm thickness. While the goal here is not to demonstrate the highest resolution possible to pattern polypeptoid brush monolayers, the successful demonstration of nanopatterns with sub-100 nm features using electron-beam lithography, together with the versatile design possible with sequence-defined polypeptoids, makes this sequence-defined polypeptoid brush platform highly attractive for applications that require incorporation of specific chemical functionalities in the desired regions.

#### Surface Chemical Contrast Nanopatterns for Selective Binding of Biomolecules

Biomolecular building blocks made of sequence-defined biomacromolecules (nucleic acids, proteins) are powerful programmable building blocks with nanometer resolution and addressability.^2^ In nanobiotechnology, bioelectronic devices often require accurate placement of these biomolecular building blocks at desired locations for device functionality.^10,11,24^ Here, we demonstrate the utility of these bioinspired, sequence-defined polypeptoid brushes as a highly versatile platform that enables precise and selective placement of biomolecular building blocks on nanopatterned surfaces.

**Figure 9:**
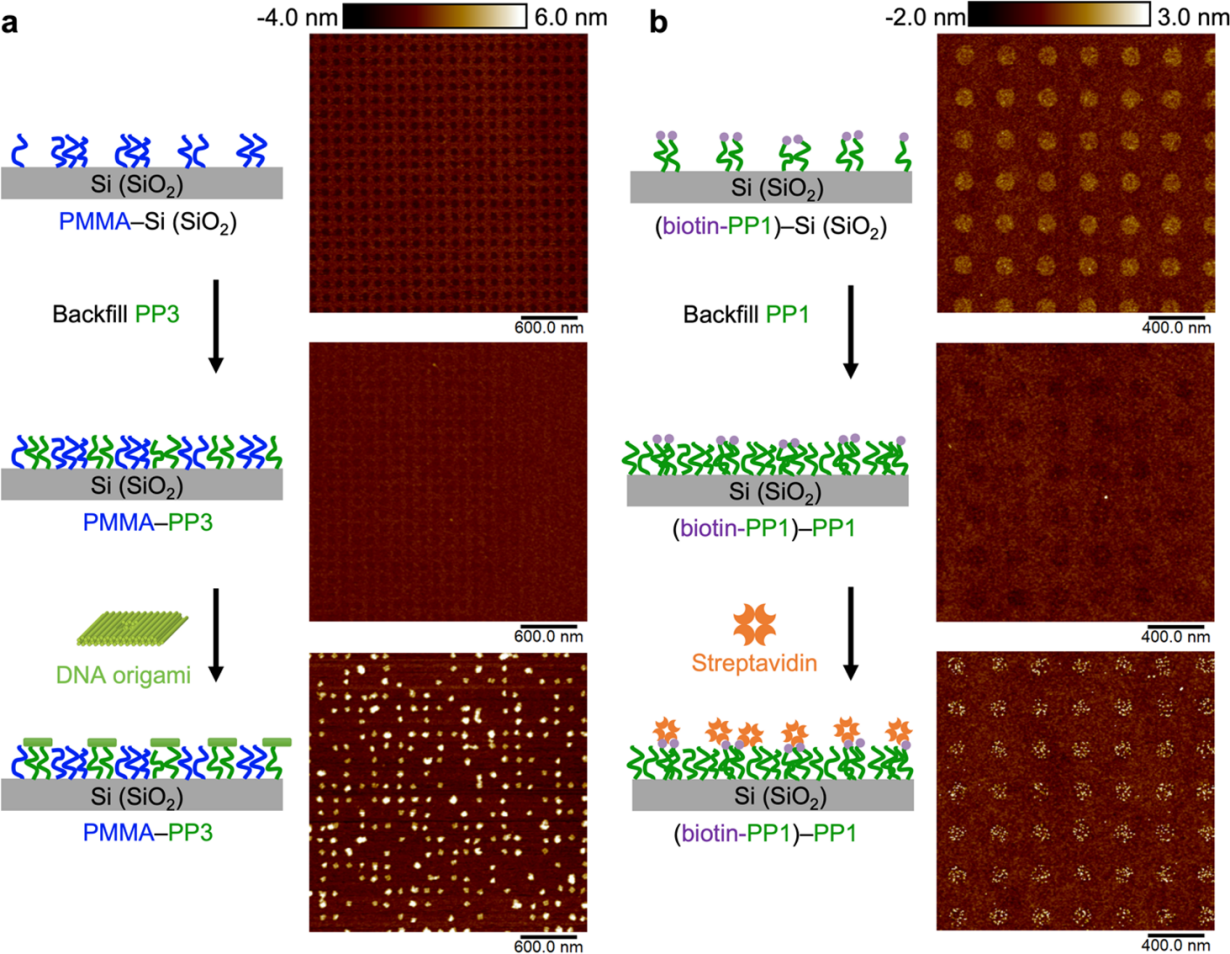
(a) Selective binding of DNA origami (61 nm × 52 nm × 8 nm cuboid nanostructure with an aperture) on surface nanopatterns of PMMA–PP**3**, where the circular patches modified by PP**3** brushes exhibit high binding affinity toward DNA origami, and the rest of the surface is passivated by PMMA brushes against DNA origami. (b) Selective binding of streptavidin (100 nM in 1× PBS buffer) on surface nanopatterns of (biotin-PP**1**)–PP**1**, where the circular patches are modified with biotin-PP**1** brushes for specific biotin–streptavidin interactions and the rest of the surface is passivated by nonbiotinylated PP**1** brushes against nonspecific binding of streptavidin.

Previous strategies for selective placement of DNA origami (in some cases with controlled orientation) on lithographically patterned Si substrates leverage electrostatic interactions, with background passivation using a hydrophobic trimethylsilyl layer produced by hexamethyldisilane (HMDS).^60−63^ Here, using the identified polypeptoid brush (PP**3**) with high DNA origami binding affinity and a PMMA brush as the surface passivation layer for the background, surface chemical contrast nanopatterns with polypeptoid-modified circular patches of commensurate feature size are fabricated for selective binding of the 61 nm × 52 nm DNA origami nanostructures (Figure 9a). Previous studies on individual DNA origami placement and orientation on lithographically patterned surfaces have reported stringent deposition and rinsing conditions.^61,62^ Here, using the PMMA–PP**3** chemical contrast nanopattern, we can achieve a relatively high binding site occupancy of individual DNA origami without complicated deposition and rinsing protocols. While achieving high individual DNA origami occupancy is not the focus of this study, we believe this polymer brush-based strategy, with robust background passivation and high yet tunable affinity rendered by sequence-defined polypeptoids, will expand the tool box for precise placement and assembly of DNA origami on nanopatterned surfaces, potentially as a more process-tolerant strategy.

Generation of well-defined, surface immobilized protein nanoarrays is important for immunoassays and pharmaceutical screening applications, as well as for proteomics research.^75,79^ Here, following a similar strategy in designing surface chemical contrast nanopatterns for selective binding, a good passivation background against streptavidin is achieved with a PP**1** brush monolayer that has minimal nonspecific binding of streptavidin, and surface regions for specific binding of streptavidin are modified by biotin-PP**1** brushes. This strategy successfully yields surface chemical contrast nanopatterns (biotin-PP**1**–PP**1**) with excellent selective immobilization of streptavidin on circular patches at length scales defined by electron-beam lithography (Figure 9b). In this case, both the background and the binding regions have minimal nonspecific binding of streptavidin, and the immobilization of streptavidin on the nanopattern is only mediated via the specific biotin–streptavidin interactions.

In this chemical contrast nanopattern design for selective immobilization of streptavidin, effects from potential insertion of the second polymer brush into the nanopatterned first polymer brush monolayer during the backfill step need to be mitigated. It is found to be necessary that the biotinylated polypeptoid brush monolayer is lithographically patterned first, and the nonbiotinylated polypeptoid brush for background passivation is backfilled as the second polymer brush; otherwise, the interdigitated biotinylated polypeptoid brush will bind a sufficient amount of streptavidin, making the background nonpassivating against streptavidin (Figure S14). This adjusted patterning strategy indicates that the covalently attached biotin functionality on polypeptoid brushes is preserved through the lithographic patterning workflow, enabling specific biotin–streptavidin binding in the final surface chemical contrast nanopatterns.

## Conclusions

In summary, we have introduced a class of surface modification materials based on bioinspired, end-grafted polypeptoid brushes. The sequence-defined synthesis of these polypeptoid brushes enables precise customization of the polypeptoid composition and sequence to match the desired functionality, processability, and biocompatibility. This material platform offers a wide spectrum of possibilities for tailoring molecular interactions at the inorganic/bio interface, ranging from simple surface energy modification (hydrophilic/hydrophobic) to various degrees of antifouling/preferential attachment properties and specific biomolecular recognition. The “grafting to” approach enhances versatility across different substrates, while compatibility with nanoscale lithographic patterning provides opportunities for engineering semiconductor/bio interfaces with single-molecule-level addressability. We demonstrated uniform, ultrathin (∼1 nm) surface modification layers capable of mediating diverse interfacial interactions through a range of polypeptoid composition and sequences. These polypeptoid brush monolayers were used to generate highly precise chemical contrast nanopatterns defined by electron-beam lithography. Importantly, we verified the preservation of chemical functionalities of the polypeptoid brushes post-lithographic patterning. By selecting appropriate polypeptoid brushes for surface passivation or target biomolecule binding, we achieved the selective immobilization of DNA origami nanostructures and streptavidin on these nanopatterns.

We believe this bioinspired, sequence-defined polypeptoid brush platform will be of interest as surface modification materials beyond the current scope of this study aimed at semiconductor/bio interfaces. The thermal stability,^32^ enzymatic resistance,^33−35^ and control over solubility make polypeptoids advantageous over natural biomacromolecules like polypeptides and nucleotides for more tolerant processing conditions, while sequence-definition renders polypeptoids the same level of programmability and precision in molecular design as biomacromolecules and potentially more flexibility in incorporation of chemical functionalities. We expect this polypeptoid brush platform could find immediate applicability in biophysical research and nanobiotechnology applications that utilize nanopatterned surfaces and structures such as for cell adhesion and signaling^77,80^ and semiconductor-biomolecule hybrid sensing systems,^4^ as well as peptide/protein sequencing.^81,82^

## Methods

### Materials

Solvents and reagents were purchased from commercial suppliers and used without further purification. Hydroxyl functionalized polystyrene (2700 g mol^–1^) and poly(methyl methacrylate) (6300 g mol^–1^) were purchased from Polymer Source (Dorval, Canada). Si substrates (prime grade, with a native oxide layer) were sourced from Addison Engineering Inc. (San Jose, CA, United States). DNA origami nanostructures (Prefabricated nanostructure PF-2, 61 × 52 × 8 nm cuboid with a 9 × 15 nm aperture, honeycomb lattice) were sourced from Tilibit Nanosystems (Munich, Germany).

### Solid-Phase Synthesis of Polypeptoids

Polypeptoids were synthesized on a custom robotic synthesizer using rink amide resin (100–200 mesh, Novabiochem) with intermediate loading (∼0.64 mmol g^–1^) and commercially available submonomers, following reported procedures.^83^ The submonomer 4-amino-1-butanol for introducing the hydroxyl group was protected by *tert*-butyldimethylsilyl (tBDMS) (Scheme S1) when it was used for solid-phase synthesis. Rink amide resin (50 μmol) was first swelled in *N*,*N*-dimethylformamide (DMF) for 10 min and deprotected with 4-methylpiperidine (1 mL, 20% v/v in DMF). Bromoacylation was performed by adding bromoacetic acid (1 mL, 0.8 M in DMF) and *N*,*N*′-diisopropylcarbodiimide (DIC) (1 mL, 0.8 M in DMF) and mixing for 20 min. Nucleophilic displacement was performed by adding the corresponding amine submonomers (1 mL, 1 M in DMF) and mixing for 1 h. The resin was washed with DMF after each synthetic step. At the end of the synthesis, the resin was washed with DMF and then with dichloromethane (DCM) and dried with a nitrogen flow.

To synthesize polypeptoids with a biotin group, biotinylation was performed on the N-terminus of polypeptoids (on resin) by coupling with d-biotin in dimethyl sulfoxide (DMSO) (Scheme S2). 16 equiv of d-biotin (0.4 M in DMSO), 16 equiv of hydroxybenzotriazole (HOBt), and 16 equiv of DIC (0.4 M in DMSO) were added to swelled resin and mixed overnight, followed by washes and drying as noted above.

Polypeptoids were cleaved from the resin using a trifluoroacetic acid (TFA) cocktail (95% TFA, 5% H_2_O) for 1 h. The typical cleavage scale is 25 μmol of resin in 3 mL of the TFA cocktail. The resin was filtered and rinsed with another 2 mL of cleavage cocktail and then rinsed with 5 mL of DCM three times. The collected solutions were dried *in vacuo* on a Biotage V10 and lyophilized from acetonitrile (ACN):H_2_O (1:1, v/v) solutions to yield the final product.

The design of polypeptoids takes into consideration crystallization inhibition. In PP**3** and PP**4** that have a long aromatic block, a monomer with phenylethyl side chain (Npe) is placed between every three monomers with phenylmethyl side chain (Npm), where the Npe monomer with one −CH_2_– longer linker serves as a crystallinity disruptor, as blocks of aromatic monomers with uniform linker length are highly crystalline and insoluble.^56^ Similarly, in PP**5**, monomers with isobutyl side chain (Nib) are used to disrupt the otherwise crystalline block of monomers with uniform *n*-butyl side chains (Nbu).^56^

### Characterization of Polypeptoids with Ultrahigh-Pressure Liquid Chromatography Mass Spectrometry (UPLC-MS)

UPLC-MS was performed on a Waters Xevo G2-XS, equipped with a time-of-flight mass spectrometer. Polypeptoid samples were dissolved at ∼0.5 mg mL^–1^ in ACN:H_2_O (1:1, v/v) and run at an eluent gradient from 5% ACN/95% H_2_O to 95% ACN/5% H_2_O (with 0.1% TFA) over 6.8 min on a C18 or C4 column.

### Preparation of Polymer Brush Monolayers Grafted on Si Substrates

Lyophilized polypeptoids with an −OH group were dissolved in dichloroethane (DCE) at 0.5 wt %, and PS–OH and PMMA–OH powders were dissolved in toluene at 1.5 wt %. Polymer solutions were filtered through 0.45, 0.2, and 0.02 μm PTFE filters. Si substrates were pretreated with UV–ozone for 5 min, and then, polymer solutions were spin coated at either 2000 rpm (polypeptoids) or 3000 rpm (PS–OH, PMMA–OH). The spin coated polypeptoid thin films were annealed at 180 °C for 30 min, and PS–OH and PMMA–OH thin films were annealed at 200 °C for 30 min, under vacuum with 10 sccm N_2_ flow. The thin films on Si substrates were then sonicated in *N*-methyl-2-pyrolidone (NMP) for 5 min, 3 times, to remove the excess ungrafted polymer chains, followed by sonication in isopropyl alcohol (IPA) for 5 min to remove the high-boiling NMP solvent. The rinsed substrates were annealed at 100 °C for 10 min under vacuum with 10 sccm N_2_ flow to remove residue solvents and leave a relaxed polymer brush monolayer grafted on the Si substrate.

### Characterization of Polymer Thin Films and Brush Monolayers on Si Substrates

Ellipsometry measurements were performed on a JA Woollam M-20000 DI ellipsometer using a Cauchy model for the polymer layer. Infrared spectroscopy measurements were performed on a Thermo-Fisher Nicolet iS50 FTIR equipped with a variable angle reflectance accessory by Harrick VariGATR and a germanium crystal. X-ray photoelectron spectroscopy (XPS) measurements were taken on a Thermo-Fisher K-Alpha Plus XPS/UPS instrument with a monochromatic Al X-ray Source (1.486 eV). Survey spectra (1350 to −10 eV) were taken with 1 eV steps; high resolution O 1s, N 1s, C 1s, and Si 2p spectra were taken with 0.1 eV steps.

### Water Contact Angle and Surface Free Energy Measurements

Measurements were performed on a KRÜSS DSA 100E tool. Surface free energy was measured and calculated using the “two-liquid geometric approach”^84−86^ with water as the polar solvent and diiodomethane as the nonpolar solvent. Static liquid contact angles are measured by capturing an image immediately after dispensing a 2 μL liquid drop on the substrate. For each substrate, 3–5 different locations were measured for liquid contact angle, with one measurement at each location.

### Electron-Beam Lithography and Generation of Surface Nanopatterns

PMMA (950 000 g/mol, 1% in chlorobenzene) was used as a positive-tone electron-beam resist. Resist was spin coated onto a Si substrate grafted with a polymer brush monolayer (polypeptoid–OH, PS–OH, or PMMA–OH) at 3000 rpm to give a thickness of ∼40 nm and then baked at 180 °C for 5 min. The line-space or circular patterns were exposed on a Raith EBPG5200 ultra high-performance electron beam lithography system at 100 kV and 2 nA beam current, with dose ranges optimized for different nanopattern designs (dose range: 1500–3000 μC cm^–2^, 700–1000 μC cm^–2^). The resist was then developed using a high contrast cold development process by sonicating in IPA/water (7:3, v/v) at 5 °C for 100 s. The exposed polymer brushes were then dry etched using oxygen plasma on an Oxford PlasmaLab 150 Inductively Coupled Etcher (ICP) with 70 W HF power, 130 W ICP power at 4 mTorr, 20 °C, with gas flows of O_2_ (30 sccm) and He (50 sccm). The remaining resist was then stripped by sonication in NMP for 5 min, 3 times, followed by sonication in IPA for 5 min to remove the high-boiling NMP solvent. The rinsed substrates were annealed at 100 °C for 10 min under vacuum with 10 sccm N_2_ flow to remove residue solvents and leave a relaxed polymer brush monolayer grafted on the Si substrate. For surface nanopatterns that consist of two polymer brushes, the second polymer brush was backfilled to the exposed Si(SiO_2_) surface by spin coating, annealing, rinsing, and reannealing, following the same procedure as preparing the first polymer brush monolayer that was patterned (note: the backfill of the second polymer brush was performed right after the resist stripping step to minimize deactivation of the silanol groups on the just exposed Si substrates).

### AFM

Atomic force microscopy (AFM) measurements were taken on a Bruker Dimension Icon AFM, with either a noncontact tapping mode or PeakForce tapping mode.

### IR PiFM

Infrared photoinduced force microscopy (IR PiFM) measurements were conducted at Molecular Vista Inc. on a Vista One microscope, with a PT277-XIR laser from Ekspla (Vilnius, Lithuaia) as the excitation source with a full tuning wavenumber range between 7143 and 541 cm^–1^ and a spectral line width of ∼3 cm^–1^. For fixed wavenumber PiFM imaging, PiF-IR spectra were first taken on the polymer brush monolayers and substrates (patterned or nonpatterned); then, the IR wavenumber with peak intensity in the corresponding IR range of the characteristic chemical functionality of the polymer/substrate was picked for PiFM imaging (polypeptoids: amide C=O stretching ∼1660 cm^–1^; PMMA: ester C=O stretching ∼1720 cm^–1^; PS: aromatic C–H bending ∼700 cm^–1^; Si substrates with a native oxide layer: Si–O–Si stretching ∼1110 cm^–1^). All images were collected with a scan speed of 0.5 Hz. PiF-IR spectra were power normalized and acquired with a sweep time of ∼13 s. Platinum–iridium-coated NCH 300 kHz noncontact cantilevers from Nanosenors (Neuchatel, Switzerland) were used for all measurements. Surface Works was used for all of the image and data processing.

### Selective DNA Origami, Streptavidin Immobilization on Surface Nanopatterns

Upon generation of the corresponding surface nanopatterns, a 150–200 μL drop (sufficiently large to cover the entire nanopatterned area) of DNA origami solution (1 nM in Tris buffer, 40 mM MgCl_2_, pH = 8.5–9) or streptavidin solution (100 nM in 1× PBS buffer) was deposited on the nanopatterned substrate. The solution drop was then incubated on the substrate in a moisturized chamber for 1 h, followed by the corresponding rinsing protocols and drying with a N_2_ stream. Rinsing protocol: DNA origami incubated samples, immerse the substrate in 20–30 mL of deionized water for 2 min, twice; streptavidin incubated samples, immerse the substrate in 20–30 mL of 1× PBS buffer for 2 min, twice. The same incubation protocol of DNA origami or streptavidin (with concentration noted in corresponding samples) on nonpatterned substrates modified with polymer brush monolayers was adopted. For nonpassivating surfaces against streptavidin, an additional deionized water rinse was used, as salt deposits from the PBS buffer occur after large amounts of streptavidin adsorb on the incubated surface area.

## Supporting Information Available

Additional information on material synthesis, polypeptoid characterization, polymer/polypeptoid thin film and monolayer characterization, surface chemical contrast nanopattern characterization, and DNA origami and streptavidin immobilization on polymer brush modified surfaces and chemical contrast nanopatterns (PDF)
