# Supplementary material for: Nanopatterned Monolayers of Bioinspired, Sequence-Defined Polypeptoid Brushes for Semiconductor/Bio Interfaces
Source: ACS Nano. 2024 Feb 27;18(10):7411–23. doi: 10.1021/acsnano.3c10204 (PMC10938923; doi:10.1021/acsnano.3c10204)
Supplement: Supplementary file 1 — nn3c10204_si_001.pdf [file nn3c10204_si_001.pdf]

Supporting Information for

Nanopatterned Monolayers of Bioinspired, Sequence-Defined Polypeptoid Brushes for Semiconductor/Bio Interfaces

Beihang Yu,[1] Boyce S. Chang,[1] Whitney S. Loo,[1,2] Scott Dhuey,[1] Padraic O'Reilly,[3] Paul D. Ashby,[1] Michael D. Connolly,[1] Grigory Tikhomirov,[4] Ronald N. Zuckermann,[1] Ricardo Ruiz[1,]*

[1]The Molecular Foundry, Lawrence Berkeley National Laboratory, Berkeley, CA 94720, USA

[2]Prizker School of Molecular Engineering, University of Chicago, Chicago, IL 60637, USA

[3]Molecular Vista Inc., San Jose, CA 95119, USA

[4]Department of Electrical Engineering and Computer Sciences, University of California, Berkeley, Berkeley, CA 94709, USA

[*]Corresponding author: R. R. (ricardo.ruiz@lbl.gov)

Table of Contents

Addition Information on Material Synthesis 3

Polypeptoid Characterization 4

Polymer/Polypeptoid Thin Film and Monolayer Characterization 12

Surface Chemical Contrast Nanopattern Characterization 14

DNA Origami and Streptavidin Immobilization on Polymer Brush Modified Surfaces and Chemical Contrast Nanopatterns 15

References 18

Addition Information on Material Synthesis

4-[(*tert*-butyldimethylsilyl)oxy]butan-1-amine. Tert-butyldimethylsilyl (tBDMS) protection of 4-amino-1-butanol was carried out following a similar protocol reported previously.[1] A solution of tert-butyldimethylsilyl chloride (tBDMS-Cl, 2.1 g, 14 mmol) in dichloromethane (DCM, 2.6 mL) was added dropwise at 0 °C to a vigorously stirred solution of 4-amino-1-butanol (5 g, 56 mmol) in DCM (1.3 mL), and stirring was continued for 1 h at 0 °C. The mixture was allowed to warm to room temperature and stirred for an additional 16–24 h. The crude mixture was poured into water (20 mL), and the layers were separated. The organic layer was washed with water (2 × 20 mL), and the aqueous layers was re-extracted with DCM (2 × 20 mL). The combined organic extracts were dried over Na_2SO_4, then the solvent was removed *in vacuo*, yielding 2.69 g (94%) product of a colorless oil.

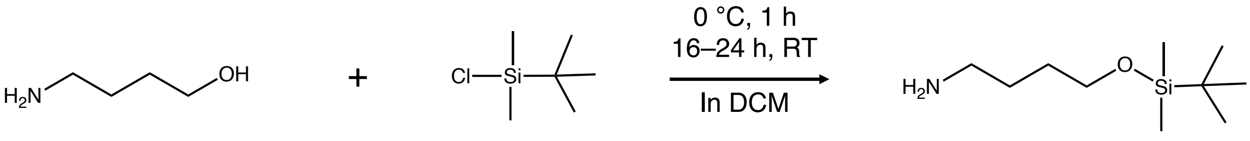

Scheme S1. Tert-butyldimethylsilyl (tBDMS) protection 4-amino-1-butanol.

Biotinylation of polypeptoids. Rink amide resin with polypeptoid (25 μmol) was re-swelled in *N*,*N*-dimethylformamide (DMF) for 20–30 min, drained. D-biotin (0.4 M, 16 equiv.) and hydroxybenzotriazole hydrate (HOBt · xH_2O, 0.4 M, 16 equiv.) in dimethyl sulfoxide (DMSO, 1 mL) were added, followed by diisopropylcarbodiimide (DIC) (0.4 M, 16 equiv.) in DMSO. The syringe reaction vessel was allowed to mix on a rocker for 20–24 h. The resin was washed with DMF, then dichloromethane (DCM), and dried with a nitrogen flow.

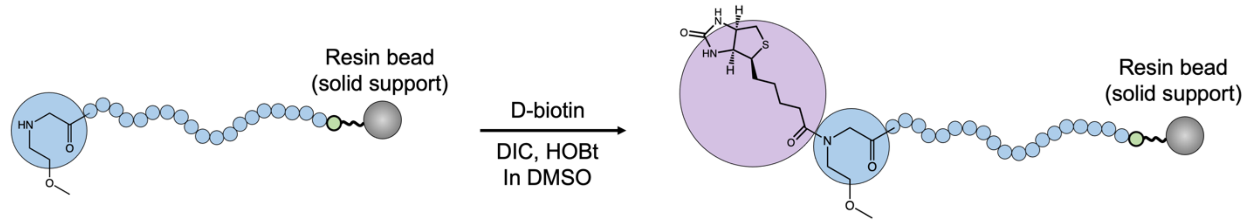

Scheme S2. Biotinylation of polypeptoids at the N-terminus.

Polypeptoid Characterization

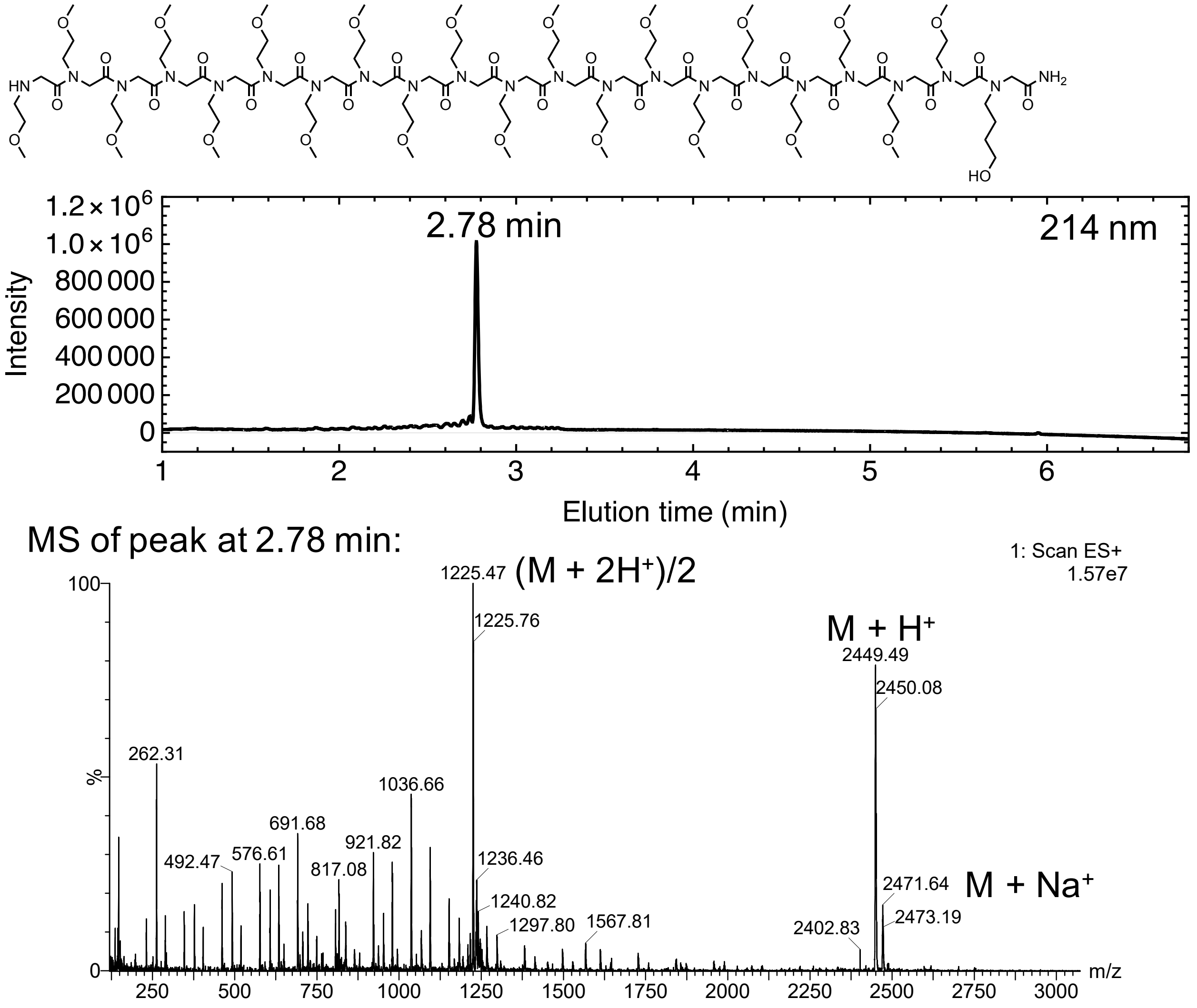

Figure S1. UPLC-MS (C18 column) of PP1, $Nme_{20}Nhb$, theoretical molecular weight: 2448.8 g mol^{-1}.

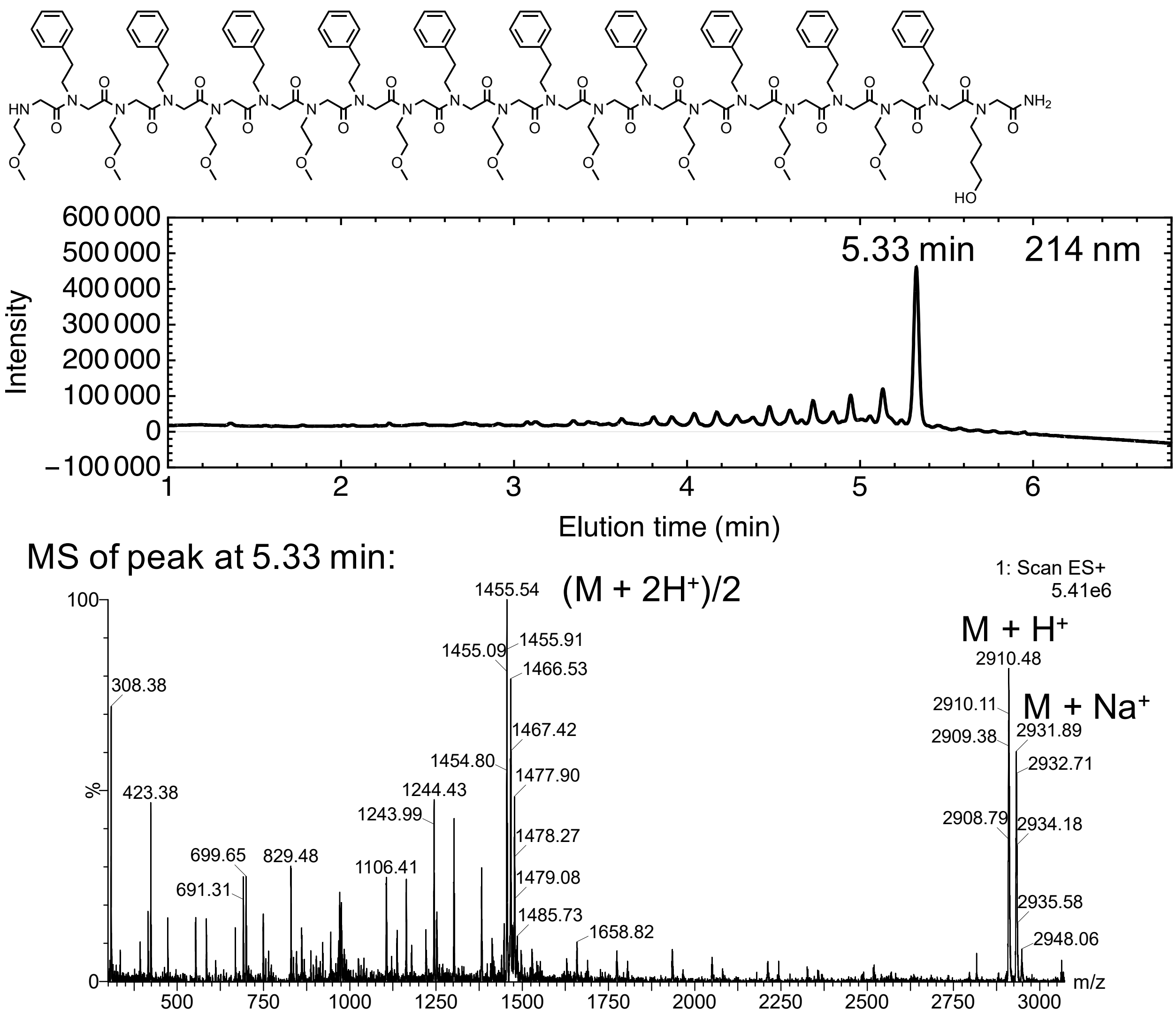

Figure S2. UPLC-MS of PP2 (C18 column), $(NmeNpe)_{10}Nhb$, theoretical molecular weight: 2909.6 g mol^{-1}.

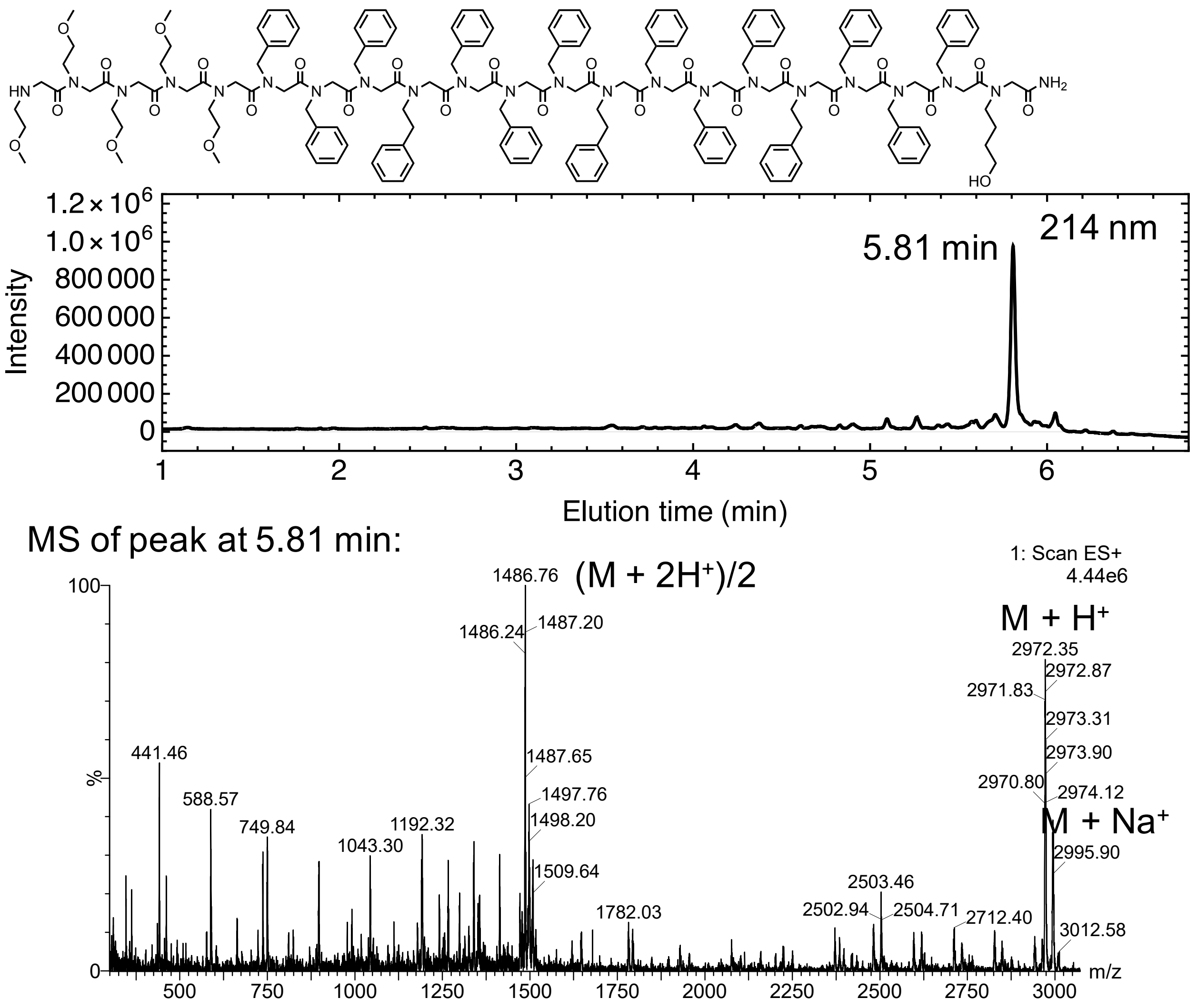

Figure S3. UPLC-MS of PP3 (C18 column), $Nme_5(Npm_3Npe)_3Npm_3Nhb$, theoretical molecular weight: 2971.6 g mol^{-1}.

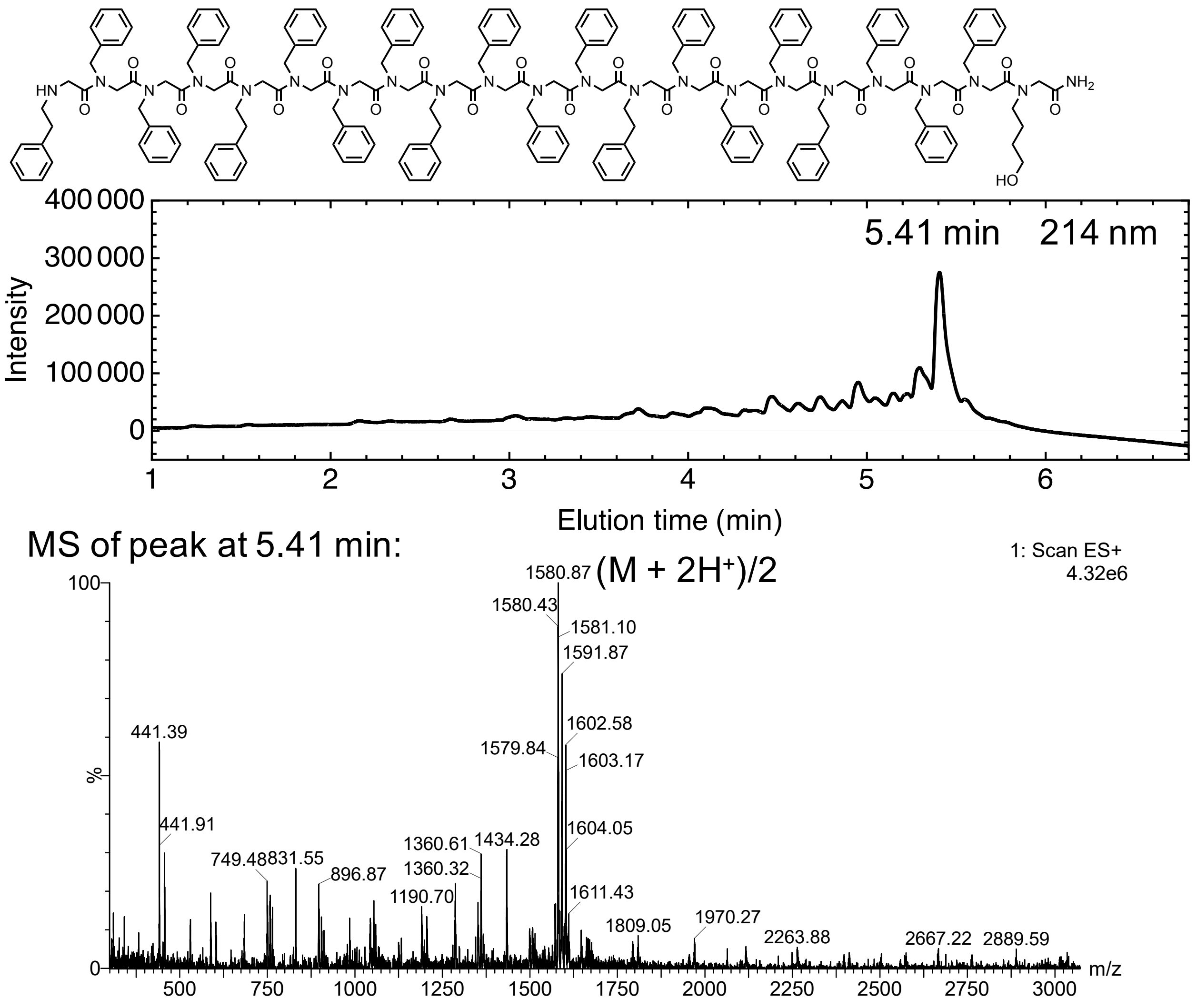

Figure S4. UPLC-MS (C4 column) of PP4, $(NpeNpm_3)_5Nhb$, theoretical molecular weight: 3159.9 g mol^{-1}.

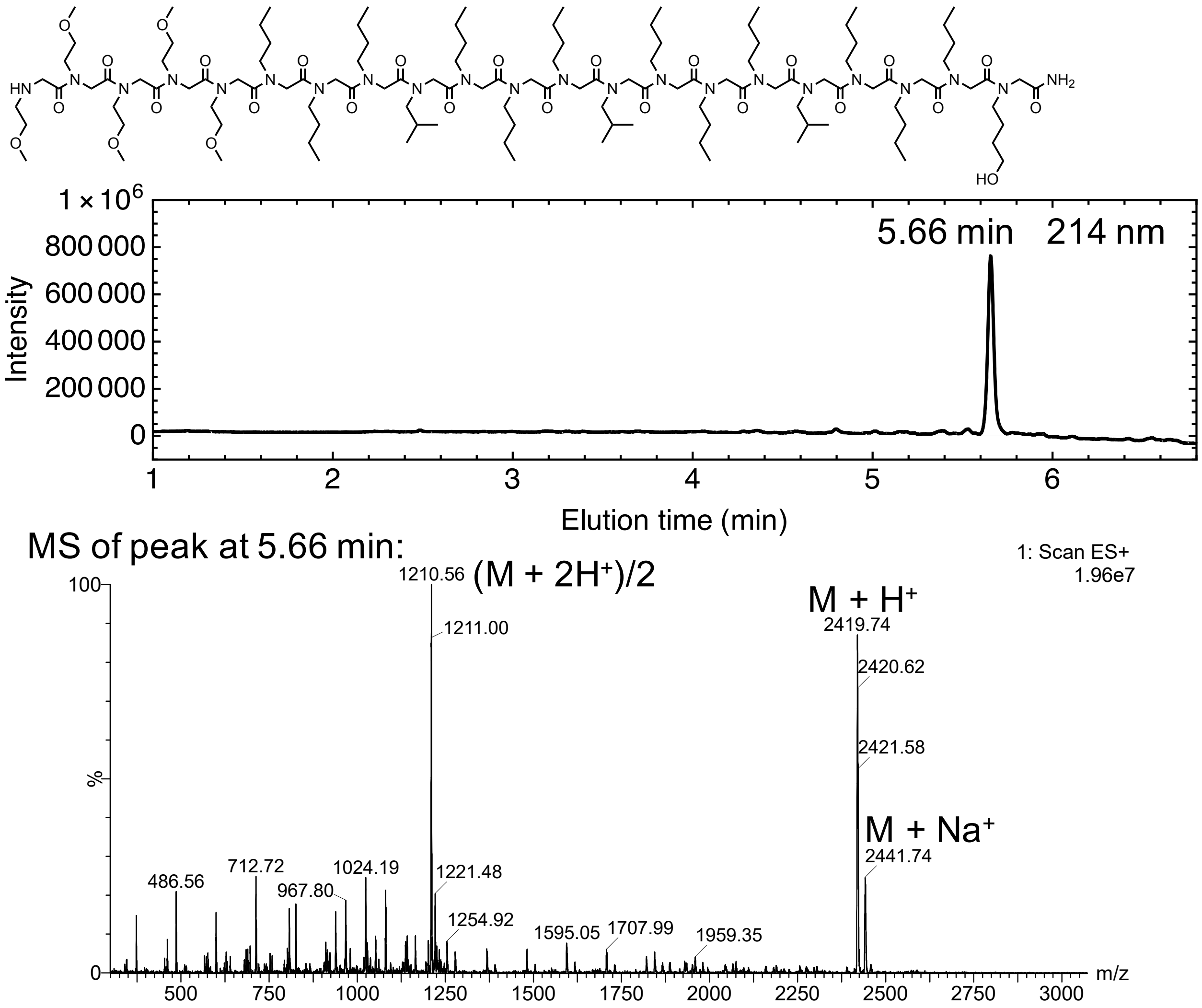

Figure S5. UPLC-MS of PP5 (C18 column), Nme$_5$(Nbu$_3$Nib)$_3$Nbu$_3$Nhb, theoretical molecular weight: 2419.3 g mol^{-1}.

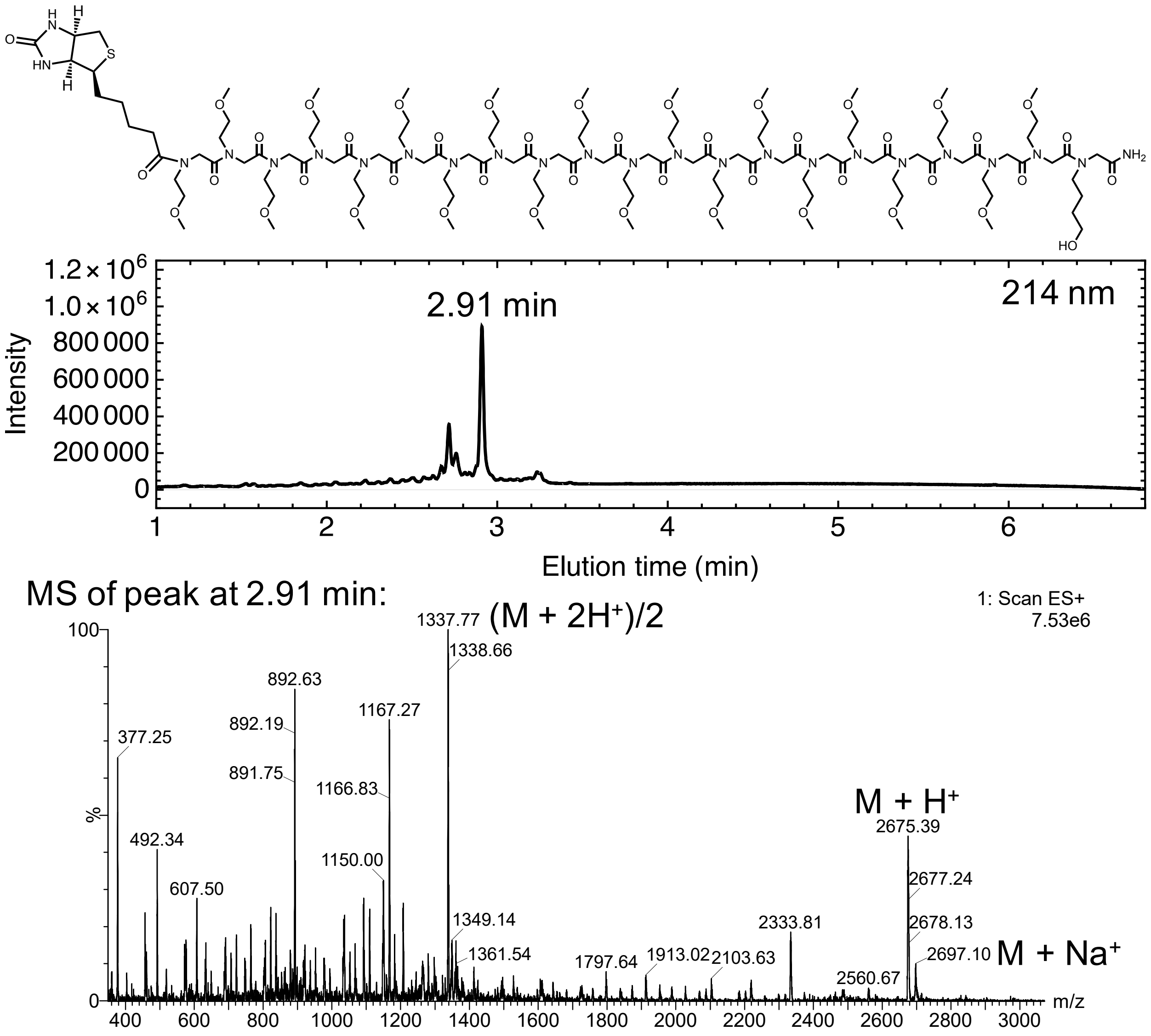

Figure S6. UPLC-MS of biotin-PP1 (C18 column), biotin-Nme_{20}Nhb, theoretical molecular weight: 2675.1 g mol^{-1}.

PP1 right after cleavage (with a potential esterification product with TFA):

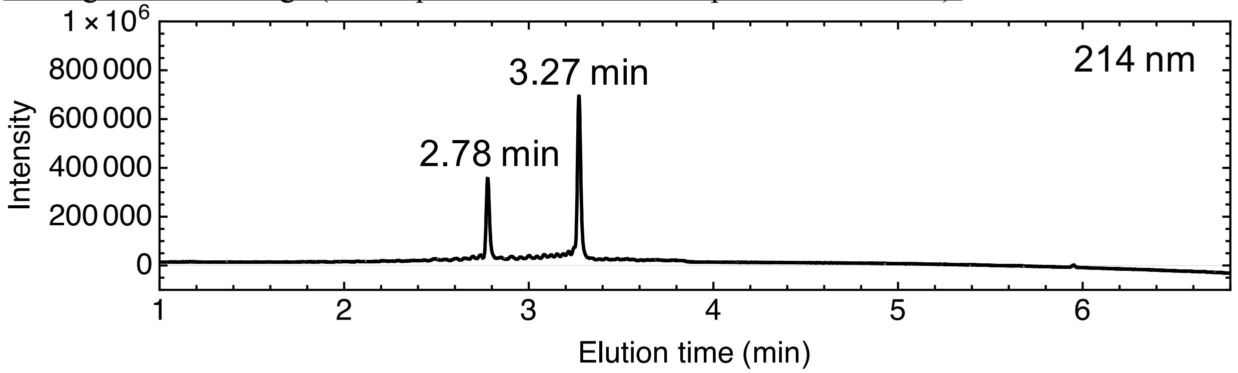

MS of peak at 2.78 min (target product):

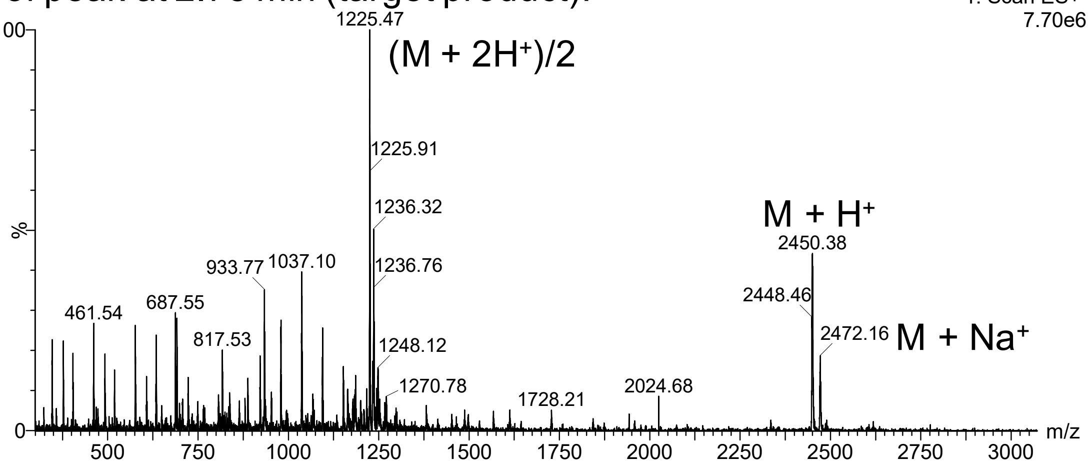

MS of peak at 3.27 min (potential esterification product):

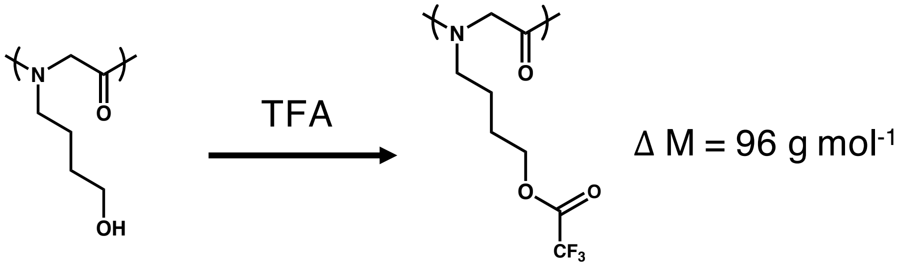

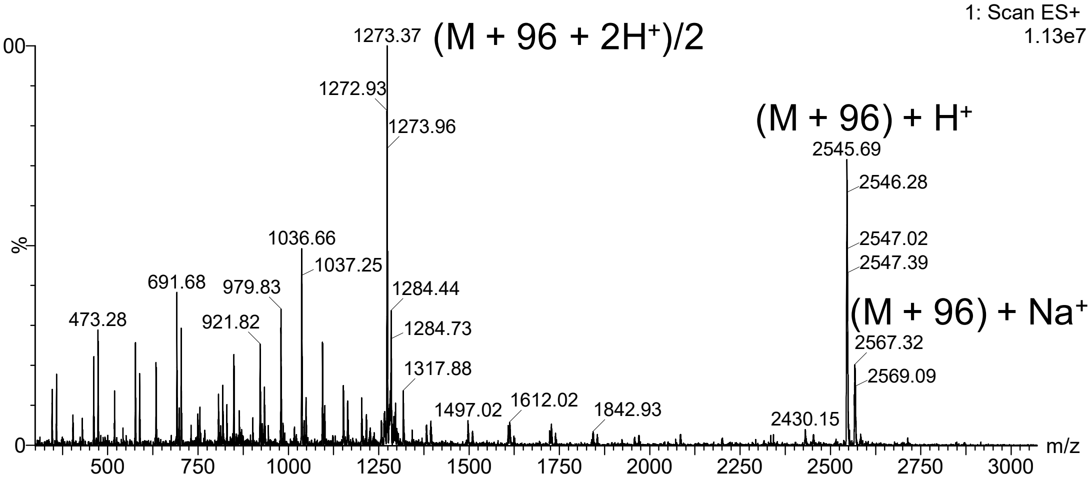

The same PP1 sample, after two days in ACN/H_2O solution:

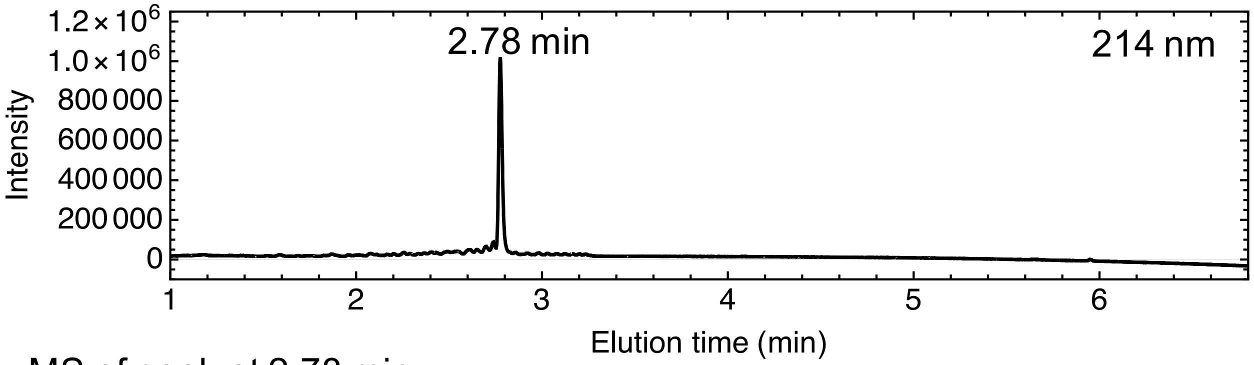

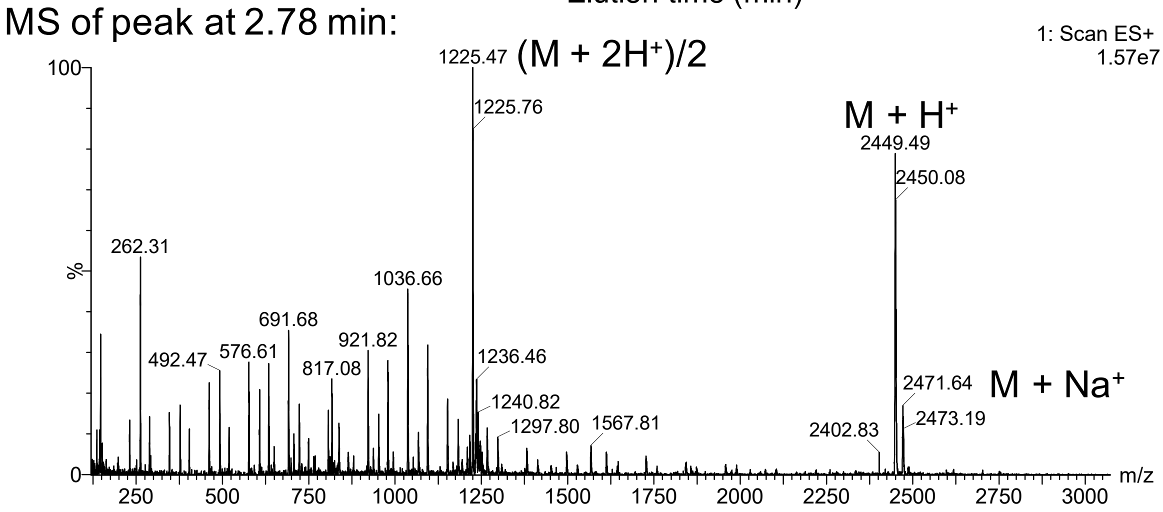

Figure S7. Potential esterification of the –OH group on the first monomer with TFA during cleavage (peak at 3.27 min observed in LC trace, with + 96 g mol^{-1} as compared to the theoretical molecular weight of PP1), which fully reverse after ~ two days in ACN/H_2O solution.

Polymer/Polypeptoid Thin Film and Monolayer Characterization

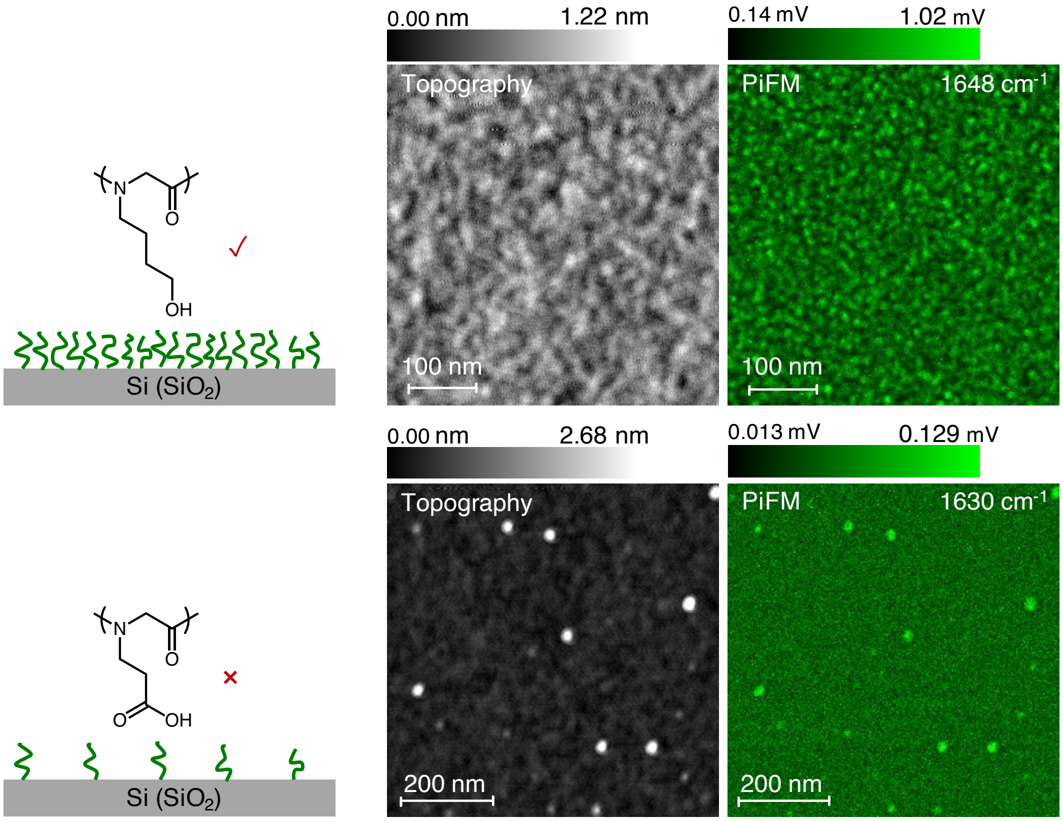

Figure S8. Topography and PiFM images of prepared polypeptoid brush monolayers on Si substrates with –OH vs. –COOH functionalized polypeptoids (Nme$_5$(Npm$_3$Npe)$_3$Npm$_3$Nhb vs. Nme$_5$(Npm$_3$Npe)$_3$Npm$_3$Nce).

Table S1. Comparison of brush monolayer thickness and water contact angle of the correspondingly modified Si substrate surface between –OH and –COOH functionalized polymers.

	M_n (g mol^{-1})	Monolayer thickness (nm)[a]	Water contact angle
PS–OH	10,000	5.70	(89.5 ± 0.1)°
PS–COOH	10,000	0.48	(56.0 ± 0.0)°
PMMA–OH	6300	3.89	(63.9 ± 0.6)°
PMMA–COOH	8400	< 0	(39.7 ± 3.9)°

[a]Polymer brush monolayers are prepared on Si wafers with a 250 nm thermal oxide layer, with the thermal oxide layer thickness measured via ellipsometry before polymer grafting.

Table S2. Surface free energy (SFE) of Si substrates modified by different polypeptoid brush monolayers.

	Water	Diiodomethane	SFE (total) (mN/m)	SFE (disperse) (mN/m)	SFE (polar) (mN/m)
PP**1**	(37.6 ± 0.3)°	(40.2 ± 0.6)°	64.8 ± 0.5	39.5 ± 0.3	25.3 ± 0.2
PP**2**	(67.6 ± 0.4)°	(35.7 ± 0.4)°	49.7 ± 0.4	41.7 ± 0.2	7.9 ± 0.2
PP**3**	(70.6 ± 0.1)°	(33.2 ± 0.1)°	49.2 ± 0.1	42.9 ± 0.1	6.3 ± 0.1
PP**4**	(72.5 ± 0.1)°	(31.0 ± 0.2)°	49.1 ± 0.1	43.8 ± 0.1	5.3 ± 0.1
PP**5**	(81.9 ± 0.5)°	(56.4 ± 0.3)°	35.5 ± 0.4	30.6 ± 0.2	4.8 ± 0.2

Surface free energy is calculated using a "two-liquid geometric approach"[2-4] based on static contact angles of two liquids, water (polar) and diiodomethane (nonpolar), measured immediately after dispensing a 2 μL drop of the liquid with an automated dispenser.

We note here that the polypeptoids are soluble in water (PP**1**) or diiodomethane (PP**2**-PP**5**) to some degree. While measurements were possible on the (insoluble) grafted brush monolayers, it was not possible to verify the trend on thicker polypeptoid films. We also note that thicker films of polystyrene were observed to be soluble in diiodomethane, which made it not possible to measure and calculate surface free energy using the water-diiodomethane pair. However, we did not find acknowledgements of the difficulty of performing the such measurements with the water-diiodomethane when the polymers are soluble in the polar or nonpolar liquids.[2-4]

Surface Chemical Contrast Nanopattern Characterization

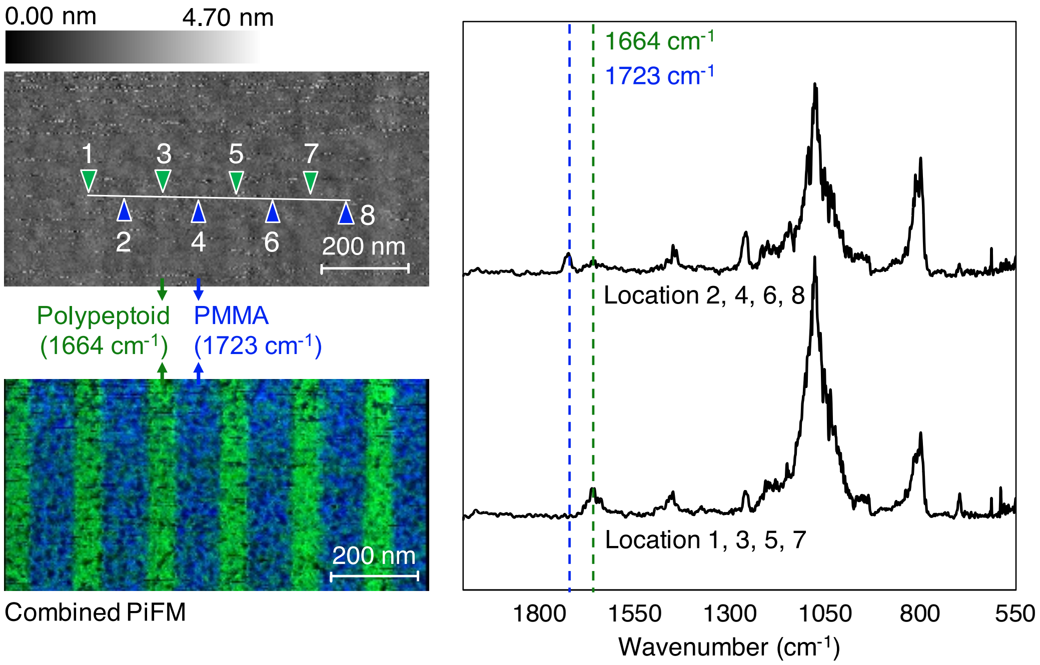

Figure S9. IR PiFM of PMMA–polypeptoid nanopattern.

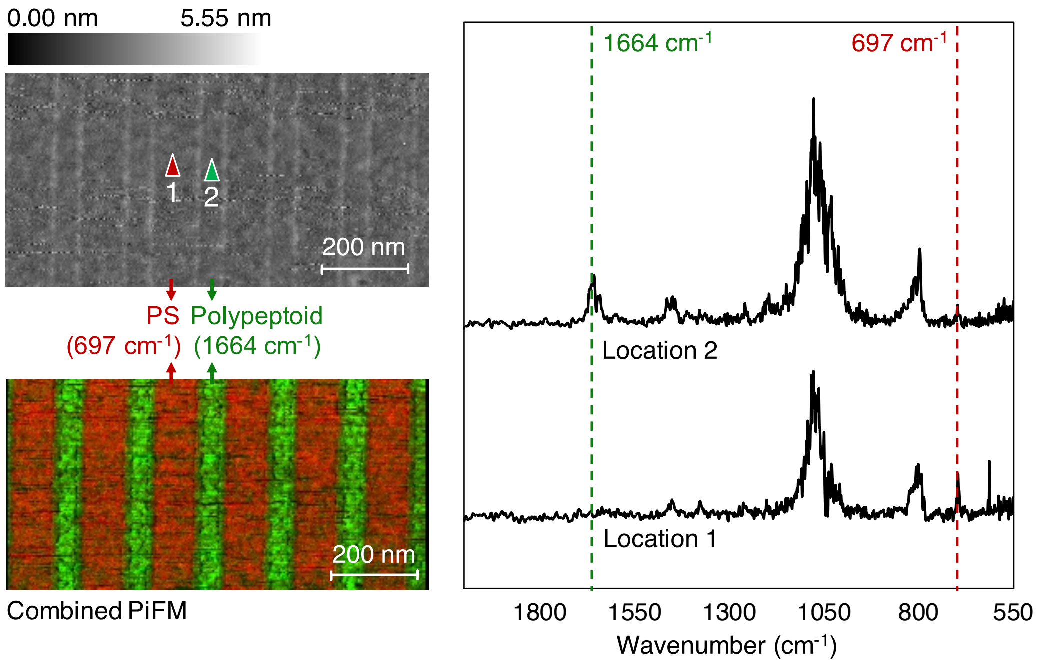

Figure S10. IR PiFM of PS–polypeptoid nanopattern.

DNA Origami and Streptavidin Immobilization on Polymer Brush Modified Surfaces and Chemical Contrast Nanopatterns

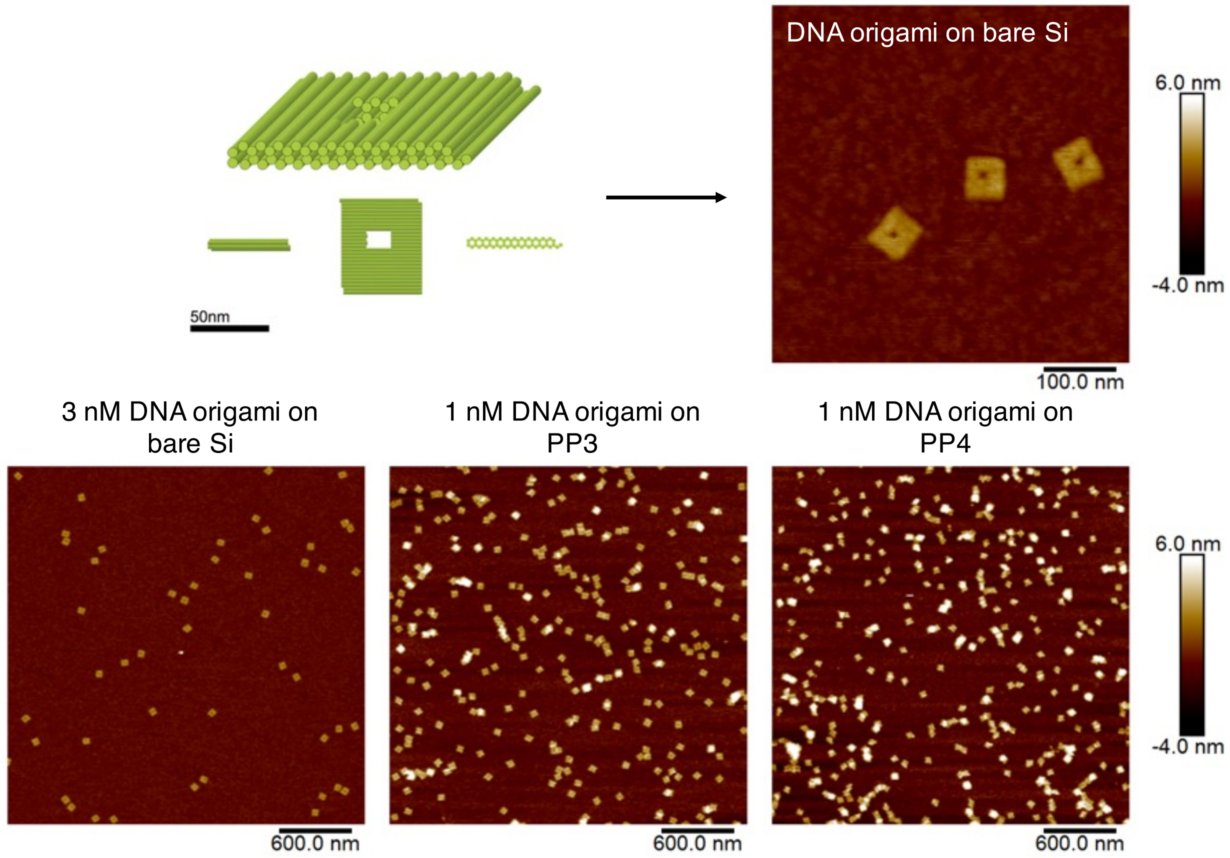

Figure S11. Top: Schematic of the DNA origami nanostructure (from tilibit nanosystems) and morphology of deposited DNA origami nanostructures on bare Si substrates. Bottom: DNA origami binding density on bare Si (3 nM) vs. on PP3 brush monolayer and PP4 brush monolayer modified Si substrates (1 nM).

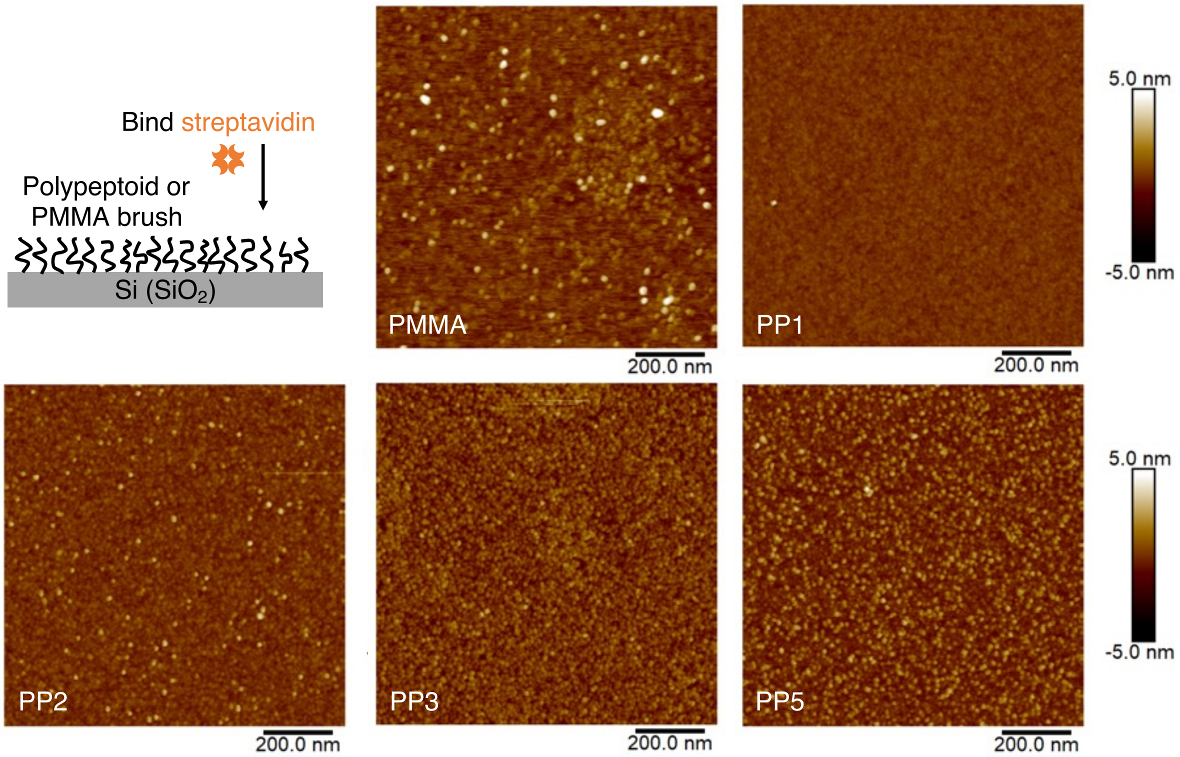

Figure S12. Passivation test against non-specific binding streptavidin (1 μM in 1× PBS buffer) on different polypeptoid brush and PMMA brush modified Si substrates.

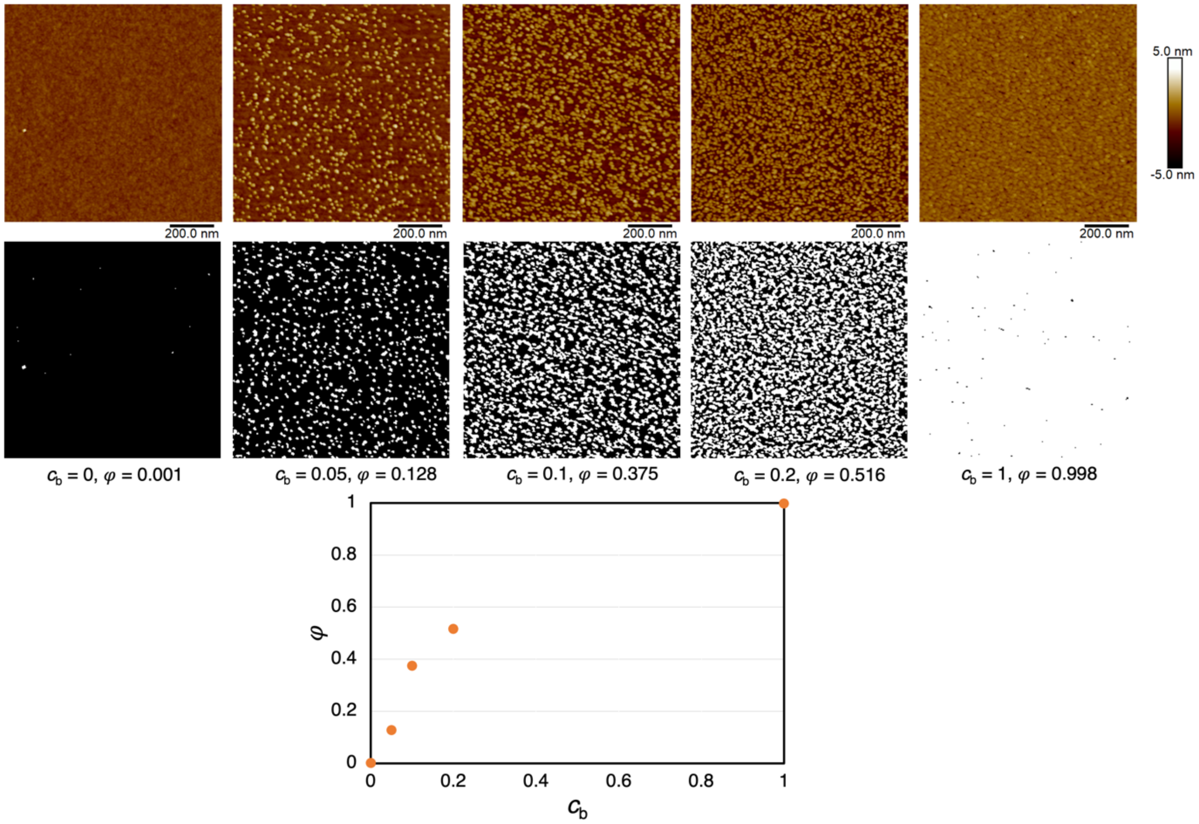

Figure S13. Different streptavidin binding density (quantified by streptavidin fractional surface coverage, φ) is achieved by controlling surface biotin group density through grafting biotin-PP**1** and PP**1** mixtures (relative concentration of biotin-PP**1** in PP**1**, c_b). Binarization of AFM images were performed by thresholding with either clustering with the "KMedoids" method (c_b = 0.05, 0.1, 0.2), or the "MinimumError" method (c_b = 0, 1) when the two height populations corresponding to streptavidin surface and polymer brush surface have minimum overlap in the height histogram.

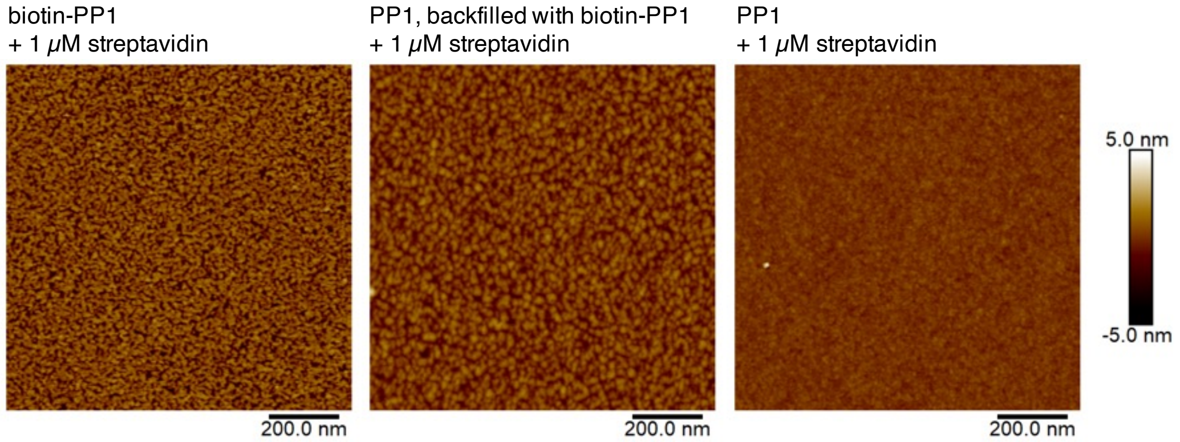

Figure S14. Backfill test with the biotinylated polypeptoid (biotin-PP1) brush, which demonstrates sufficient brush interpenetration happens during the backfill step, and the interpenetrated biotinylated polypeptoid brushes bind large amounts of streptavidin proteins.

References

1. Zuckermann, R. N.; Martin, E. J.; Spellmeyer, D. C.; Stauber, G. B.; Shoemaker, K. R.; Kerr, J. M.; Figliozzi, G. M.; Goff, D. A.; Siani, M. A.; Simon, R. J.; Banville, S. C.; Brown, E. G.; Wang, L.; Richter, L. S.; Moos, W. H., Discovery of Nanomolar Ligands for 7-Transmembrane G-Protein-Coupled Receptors from a Diverse N-(Substituted)glycine Peptoid Library. *J. Med. Chem.* **1994,** *37*, 2678-2685.
2. Owens, D. K.; Wendt, R. C., Estimation of the surface free energy of polymers. *J. Appl. Polym. Sci.* **1969,** *13*, 1741-1747.
3. Kaelble, D. H.; Uy, K. C., A Reinterpretation of Organic Liquid-Polytetrafluoroethylene Surface Interactions. *J. Adhes.* **1970,** *2*, 50-60.
4. Kaelble, D. H., Dispersion-Polar Surface Tension Properties of Organic Solids. *J. Adhes.* **1970,** *2*, 66-81.
